# Repurposed Fenoprofen Targeting SaeR Attenuates *Staphylococcus aureus* Virulence in Implant-Associated Infections

**DOI:** 10.1021/acscentsci.3c00499

**Published:** 2023-06-15

**Authors:** Feng Jiang, Yingjia Chen, Jinlong Yu, Feiyang Zhang, Qian Liu, Lei He, Hamushan Musha, Jiafei Du, Boyong Wang, Pei Han, Xiaohua Chen, Jin Tang, Min Li, Hao Shen

**Affiliations:** † Department of Orthopedics, Shanghai Sixth People’s Hospital Affiliated to Shanghai Jiao Tong University School of Medicine, Shanghai 200235, China; ‡ Drug Discovery and Design Center, State Key Laboratory of Drug Research, Shanghai Institute of Materia Medica, Chinese Academy of Sciences, 555 Zuchongzhi Road, Shanghai 201203, China; § Department of Pharmacy, University of Chinese Academy of Sciences, No.19A Yuan Road, Beijing 100049, China; ∥ Department of Laboratory Medicine, Renji Hospital, School of Medicine, Shanghai Jiaotong University, Shanghai 200127, China; ⊥ Department of Clinical Laboratory, Shanghai Sixth People’s Hospital Affiliated to Shanghai Jiao Tong University School of Medicine, Shanghai 200235, China; # Faculty of Medical Laboratory Science, Shanghai Jiaotong University School of Medicine, Shanghai 200025, China; ▽ Department of Infectious Diseases, Shanghai Sixth People’s Hospital Affiliated to Shanghai Jiao Tong University School of Medicine, Shanghai 200235, China

## Abstract

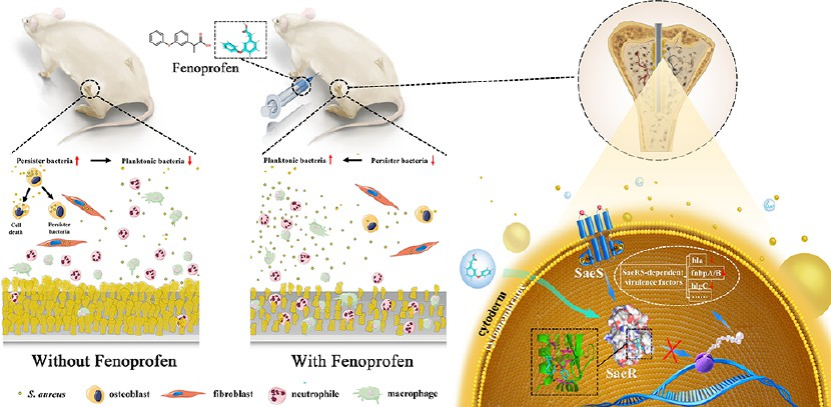

Implant-associated infections (IAIs) caused by *S. aureus* can result in serious challenges after orthopedic surgery. Due to biofilm formation and antibiotic resistance, this refractory infection is highly prevalent, and finding drugs to attenuate bacterial virulence is becoming a rational alternative strategy. In *S. aureus*, the SaeRS two-component system (TCS) plays a key role in the production of over 20 virulence factors and the pathogenesis of the bacterium. Here, by conducting a structure-based virtual screening against SaeR, we identified that fenoprofen, a USA Food and Drug Administration (FDA)-approved nonsteroid anti-inflammatory drug (NSAID), had excellent inhibitory potency against the response regulator SaeR protein. We showed that fenoprofen attenuated the virulence of *S. aureus* without drug resistance. In addition, it was helpful in relieving osteolysis and restoring the walking ability of mice in vitro and in implant-associated infection models. More importantly, fenoprofen treatment suppressed biofilm formation and changed the biofilm structure, which caused *S. aureus* to form loose and porous biofilms that were more vulnerable to infiltration and elimination by leukocytes. Our results reveal that fenoprofen is a potent antivirulence agent with potential value in clinical applications and that SaeR is a drug target against *S. aureus* implant-associated infections.

## Abstract 2

Implant-associated infections (IAIs) caused by *S. aureus* can result in serious challenges after orthopedic surgery. Due to biofilm formation and antibiotic resistance, this refractory infection is highly prevalent, and finding drugs to attenuate bacterial virulence is becoming a rational alternative strategy. In *S. aureus*, the SaeRS two-component system (TCS) plays a key role in the production of over 20 virulence factors and the pathogenesis of the bacterium. Here, by conducting a structure-based virtual screening against SaeR, we identified that fenoprofen, a USA Food and Drug Administration (FDA)-approved nonsteroid anti-inflammatory drug (NSAID), had excellent inhibitory potency against the response regulator SaeR protein. We showed that fenoprofen attenuated the virulence of *S. aureus* without drug resistance. In addition, it was helpful in relieving osteolysis and restoring the walking ability of mice in vitro and in implant-associated infection models. More importantly, fenoprofen treatment suppressed biofilm formation and changed the biofilm structure, which caused *S. aureus* to form loose and porous biofilms that were more vulnerable to infiltration and elimination by leukocytes. Our results reveal that fenoprofen is a potent antivirulence agent with potential value in clinical applications and that SaeR is a drug target against *S. aureus* implant-associated infections.

## Introduction

Implant-associated infections (IAIs) cause severe complications in orthopedics, and two-thirds of IAIs are caused by *S. aureus*.^1,2^ In addition to secreting a large amount of virulent substances that cause serious infection, *S. aureus* can survive by forming biofilms on implant surfaces and by invading nonprofessional phagocytes to evade host immune defenses and antibiotics.^3−7^ Treating IAIs is especially difficult, as *S. aureus* can adapt to changing environments. Therefore, finding innovative but feasible methods to prevent and treat orthopedic *S. aureus* IAIs is particularly important.

Due to the overuse of antibiotics and the subsequent prevalence of multidrug-resistant strains, especially methicillin-resistant *S. aureus* (MRSA), the WHO recommends that *S. aureus* should be a high priority in drug development efforts.^8^ A promising strategy involves the design of antivirulence drugs that disrupt the expression of virulence factors but not the growth or viability of pathogens. Antivirulence agents do not impose strong selective pressures on bacteria that favor the evolution of resistance and persistence mechanisms, which ultimately decrease the occurrence of antibiotic resistance.^9^ In addition, antivirulence drugs attenuate the expression of virulence factors and thus neutralize virulence-mediated immune evasion and suppression.^10,11^

The differences in the in vivo pathogenic processes and adaptability of *S. aureus* are largely attributed to its ability to coordinate the expression and secretion of toxins, adhesive proteins, and other virulence factors based on changing environmental stimuli, and this ability is predominantly mediated by its general regulators, the two-component systems (TCSs).^12−14^ These TCSs are attractive targets for novel antivirulence agents and have recently aroused great interest.^15−17^ One of the best studied is AgrAC TCS, which is activated in response to bacterial density and promotes the expression of virulence factors, contributing to toxin-mediated pathogenesis.^18^ Dysfunctional mutations of the agr system help to suppress *S. aureus* skin and intestinal colonization,^19,20^ which are nonimplant-associated infections. However, we recently found that *S. aureus* agr-dysfunctional mutants could be isolated from clinical cases of surgical implant-associated infection^21^ and that prolonged infection increased the rate of agr-dysfunctional mutant development. Our previous in vivo observations revealed that agr-dysfunctional isolates could form robust and enlarged biofilms on the implant surface to increase the resistance of agr-dysfunctional biofilms to leukocyte attacks.^22^ The distinct roles of Agr TCSs in biofilm- and nonbiofilm-associated infections suggest that *S. aureus* infection is a dynamic process and that the two-component systems have different adaptive effects for various types of infections. Therefore, looking for a TCS target that could be useful in implant-associated infections and developing effective drugs are promising to improve therapeutic strategies against *S. aureus* implant-associated infections.

Among the TCSs in *S. aureus*, the SaeRS TCS plays an important role in regulating the expression of virulence factors and has been shown to be involved in bacterial biofilm formation.^23,24^ SaeRS TCS is composed of the sensor histidine kinase SaeS, response regulator SaeR, and two auxiliary proteins SaeP and SaeQ.^25^ The sensor histidine kinase SaeS senses environmental signals and autophosphorylates; then, phosphorylated SaeR (SaeR-P) binds to the SaeR binding sequence (SBS) to activate the transcription of SaeP, SaeQ, and over 20 virulence factors.^26^ Xanthoangelol B1 and its derivative PM-56 were previously shown to bind to SaeS and can thus inhibit its histidine kinase activity.^27^ Whereas suppressing saeS may be unreliable and incomplete, saeR may still be phosphorylated by some other unknown signals and then activate the expression of downstream regulated virulence genes.^26^ To our knowledge, there is currently no study on drugs that directly bind the functional domain of the SaeR protein, which could significantly inhibit the transcriptional regulatory function of the SaeR protein to downstream virulence factors.

Previous research on SaeRS TCSs in infection has mostly focused on nonbiofilm infections, such as skin infection, bloodstream infection, and osteomyelitis,^28−30^ while research on implant-associated biofilm infections in vivo is relatively lacking. In this study, we first verified the importance of the SaeRS TCS in the pathogenicity of *S. aureus* IAIs in vivo, and then structure-based virtual screening was used to screen for inhibitors of the SaeR protein from the DrugBank database. A group of nonsteroidal anti-inflammatory drugs (NSAIDs) were among the drug candidates that attracted our attention. NSAIDs are recommended for pain relief in the perioperative period after orthopedic surgery,^31−33^ and they have been reported to have an anti-infection effect, but the mechanism remains unclear.^34^ Therefore, we selected NSAIDs and then verified their efficiency in inhibiting the SaeR protein by using the GFP reporter system. We identified fenoprofen, an FDA-approved nonsteroidal anti-inflammatory drug, as an inhibitor of the SaeR protein. Subsequently, we demonstrated that fenoprofen can bind to the functional domain of SaeR protein, which prevents the transcription of virulence factors from being activated, ultimately leading to suppressed *S. aureus* virulence and accelerated recovery from infection. Furthermore, we found that fenoprofen attenuated the osteoblast internalization of *S. aureus* and caused the *S. aureus* biofilms on implant surfaces to become sparse and porous, which made it easier for the host immune system to clear planktonic and adherent bacteria from the infected site. More importantly, these effects of fenoprofen were effective against all of the clinical strains isolated from orthopedic implant-associated infections, including both MSSA and MRSA. In addition, no drug resistance in *S. aureus* was observed after continuous treatment with fenoprofen.

In our research, we proposed and confirmed the competitive inhibitory effect of fenoprofen on the *S. aureus* SaeR protein for the first time, illustrated a novel NSAID antibacterial mechanism, and revealed a new potential of transforming traditional NSAIDs, fenoprofen for perioperative prevention and treatment of *S. aureus* infections in orthopedics, for clinical applications.

## Results

### SaeRS TCS is Important for Pathogenesis in Implant-Associated *S. aureus* Biofilm Infection

**Figure 1:**
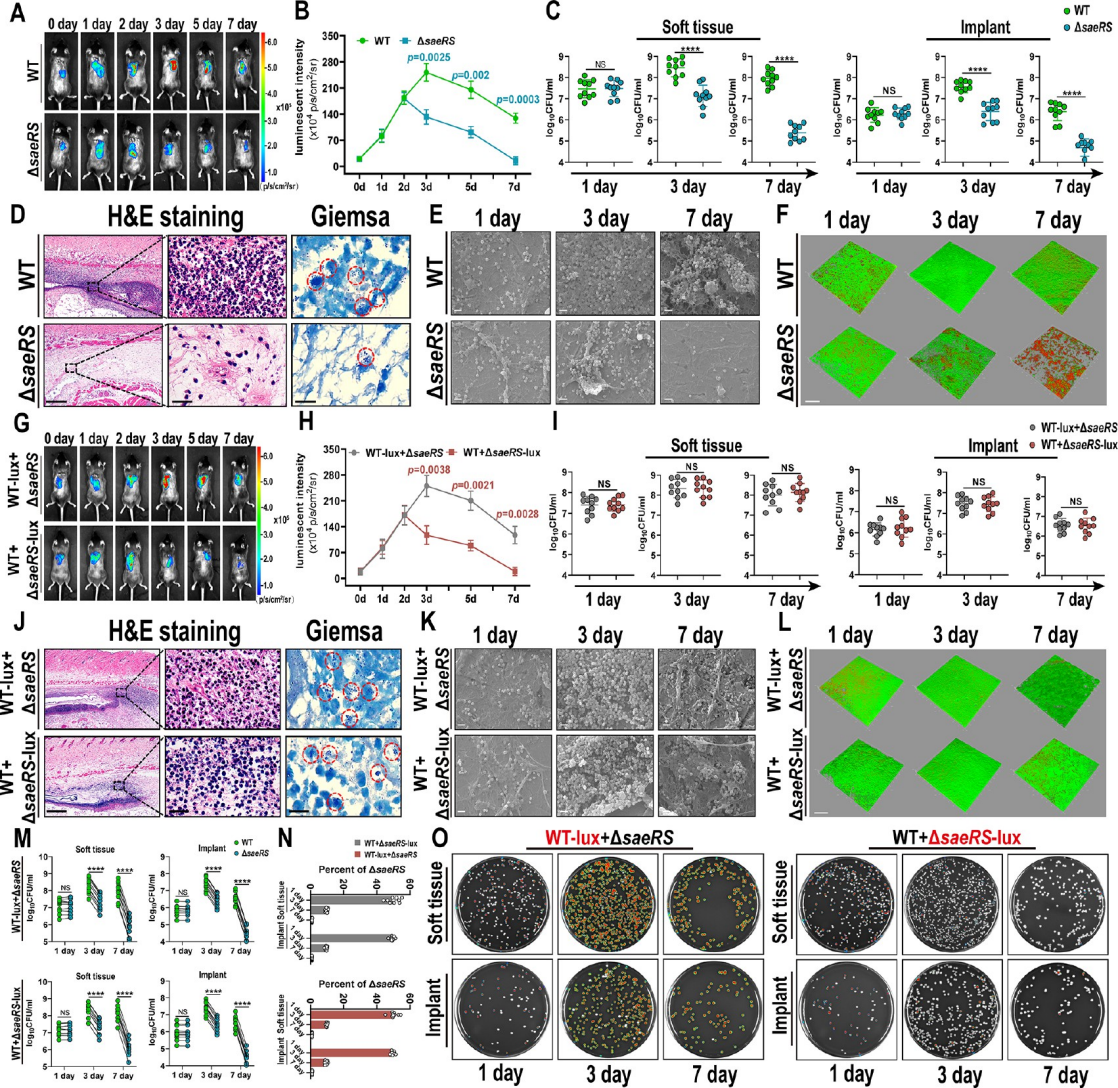
SaeRS TCS is important for pathogenesis in implant-associated biofilm infection. (A,B) Representative bioluminescence images and intensity of the implant-associated biofilm infection model at days 0, 1, 2, 3, 5, and 7. The color bar indicates the relative bacterial photon intensity (p/s/cm2/sr) (*n* = 3 for each time point). (C) CFU counting results of the implant and surrounding infected soft tissue in the implant-associated *S. aureus* infection model at days 1, 3, and 7 (*n* = 10; data are presented as individual points). (D) H&E and Giemsa staining images of the implants surrounding infected soft tissue in the implant-associated *S. aureus* infection model at day 7. Red circles indicate the bacterial residue in the soft tissue. (E,F) SEM and CLSM images of biofilms on the implant surface in implant-associated biofilm infection at days 1, 3, and 7. Live bacteria were stained green (SYTO9), and dead bacteria were stained red (PI). (G,H) Representative bioluminescence images and intensity of the mixed implant-associated biofilm infection model at days 0, 1, 2, 3, 5, and 7 (*n* = 3 for each time point). (I) Bacterial CFU counting analysis of the implant and surrounding infected soft tissue in the WT-lux/Δ*saeRS* and WT/Δ*saeRS*-lux groups at days 1, 3, and 7 (*n* = 10; data are presented as individual points). (J) H&E and Giemsa staining images of the implants surrounding infected soft tissue in the mixed implant-associated biofilm infection at day 7. Red circles indicate the bacterial residue in the soft tissue. (K,L) SEM and CLSM images of implant biofilms in the WT-lux/Δ*saeRS* and WT/Δ*saeRS*-lux groups at days 1, 3, and 7. Live bacteria were stained green (SYTO9), and dead bacteria were stained red (PI). (M–O) Representative culture images (O) and corresponding bacterial counting results (M and N) of bacterial colonies from implants and surrounding tissues at days 1, 3, and 7 after infection (*n* = 10; data are presented as individual points). The lux strain showed bioluminescence. Scale bars, 500 and 50 μm in H&E, 10 μm in Giemsa (D and J), 5 μm in (E and K), and 200 μm in (F and L). All results are presented as the means ± SD ****P < 0.0001, and data were analyzed by two-way ANOVA with Tukey’s multiple comparison post-tests (B and H), two-tailed unpaired *t* tests (C and I), and two-tailed paired *t* tests (M).

To understand the importance of SaeRS TCS for pathogenesis in implant-associated biofilm infection, we first compared the virulence expression of wild-type (WT) and *saeRS* mutant strains of clinical *S. aureus* ST1792, which was isolated from the prosthesis of a periprosthetic joint infection (PJI) patient. The qPCR results agreed with the knowledge that the SaeRS TCS regulated numerous virulence factors (Figure S2A). In addition, the *saeRS* mutant showed substantially weaker hemolytic activity than the WT, which may be related to the decreased expression of *saeRS*-regulated toxins (Figure S2B). Next, we used luminescent wild-type and isogenic *saeRS* mutant strains to construct a mouse implant-associated infection model. The bacterial luminescence of the *saeRS* mutant group increased during the initial colonization stage (day 1 to day 2), peaked at day 2, and then began to decrease. Furthermore, from day 3 to day 7, the luminescence intensity of the *saeRS* mutant group was significantly weaker than that of the WT group (Figure 1A,B and Figure S3A). Further analysis of the bacterial burden revealed that from day 3 to day 7, the CFU count on the implant and surrounding soft tissue in the *saeRS* mutant group decreased by 1.71 log_10_ CFU/ml and 1.73 log_10_ CFU/ml, respectively, while that in the WT group decreased by only 0.5 log_10_ CFU/ml and 1.18 log_10_ CFU/ml, respectively, indicating that the *saeRS* mutant strain was more easily eliminated by the host (Figure 1C). Hematoxylin and eosin (H&E) and Giemsa staining at day 7 also showed mild inflammatory exudation and tissue necrosis, as well as less residual bacteria in the *saeRS* mutant group (Figure 1D). To assess the dynamics of biofilms, scanning electron microscopy (SEM) and confocal laser scanning microscopy (CLSM) were used to observe the structure of biofilms at days 1, 3, and 7. Notably, the biofilm formed by the *saeRS* mutant strain on the implant surface in vivo was more vulnerable to disruption by the host, while the biofilm formed by the WT strain was still dense and intact at day 7 (Figure 1E,F). These results indicated that the *saeRS* mutant strain was more efficiently eliminated by the host during the elimination stage of implant-associated *S. aureus* infections, primarily due to its inability to form a dense mature biofilm on the implant, making it unable to resist attacks from the host immune system.

To further explore the role of the SaeRS TCS in implant-associated infections, we used mixed WT/*saeRS* mutant (1:1) inocula to generate a biofilm-associated infection model, and we designed two groups, WT-lux/Δ*saeRS* and WT/Δ*saeRS*-lux groups, which were useful to distinguish WT and *saeRS* mutant strains by whether they were luminescent strains or not (Figure S3B). In in vivo mixed infection, the bioluminescence intensity of the *saeRS* mutant strain was lower than that of the WT strain during the same period from day 3 to day 7 (Figure 1G,H). There was no significant difference in the total CFU count or pathological results between the two groups (Figure 1I,J). The biofilms on the implant surface in the two groups were also similar to those in the WT group, indicating that the *saeRS* mutant strain had no obvious effect on the survival and biofilm formation of the WT strain under the coexistence condition (Figure 1K,L). Then, we observed the fate of *saeRS* mutants by calculating the WT/mutant percentages in the implant and surrounding soft tissue at days 1, 3, and 7 after infection. In the WT-lux/Δ*saeRS* group, at day 1, the bacterial burdens of the WT and *saeRS* mutant strains remained equal. Notably, at day 3, the *saeRS* mutant percentage in the soft tissue and implant dropped from 50.2% to 9.75% ± 0.62% and from 49.37% to 9.45% ± 0.47%, respectively. At day 7, the *saeRS* mutant percentages in the soft tissue and implant were 0.90 ± 0.07% and 0.87 ± 0.07%, respectively (Figure 1M–O). The results were similar in the WT/Δ*saeRS*-Lux group (Figure 1M–O). These outcomes implied that WT bacteria were much more capable of surviving than *saeRS* mutant bacteria and indicated that the SaeRS TCS is very important for pathogenesis in implant-associated infection.

### Fenoprofen Was Screened in Silico As an Inhibitor of the SaeR Protein

**Figure 2:**
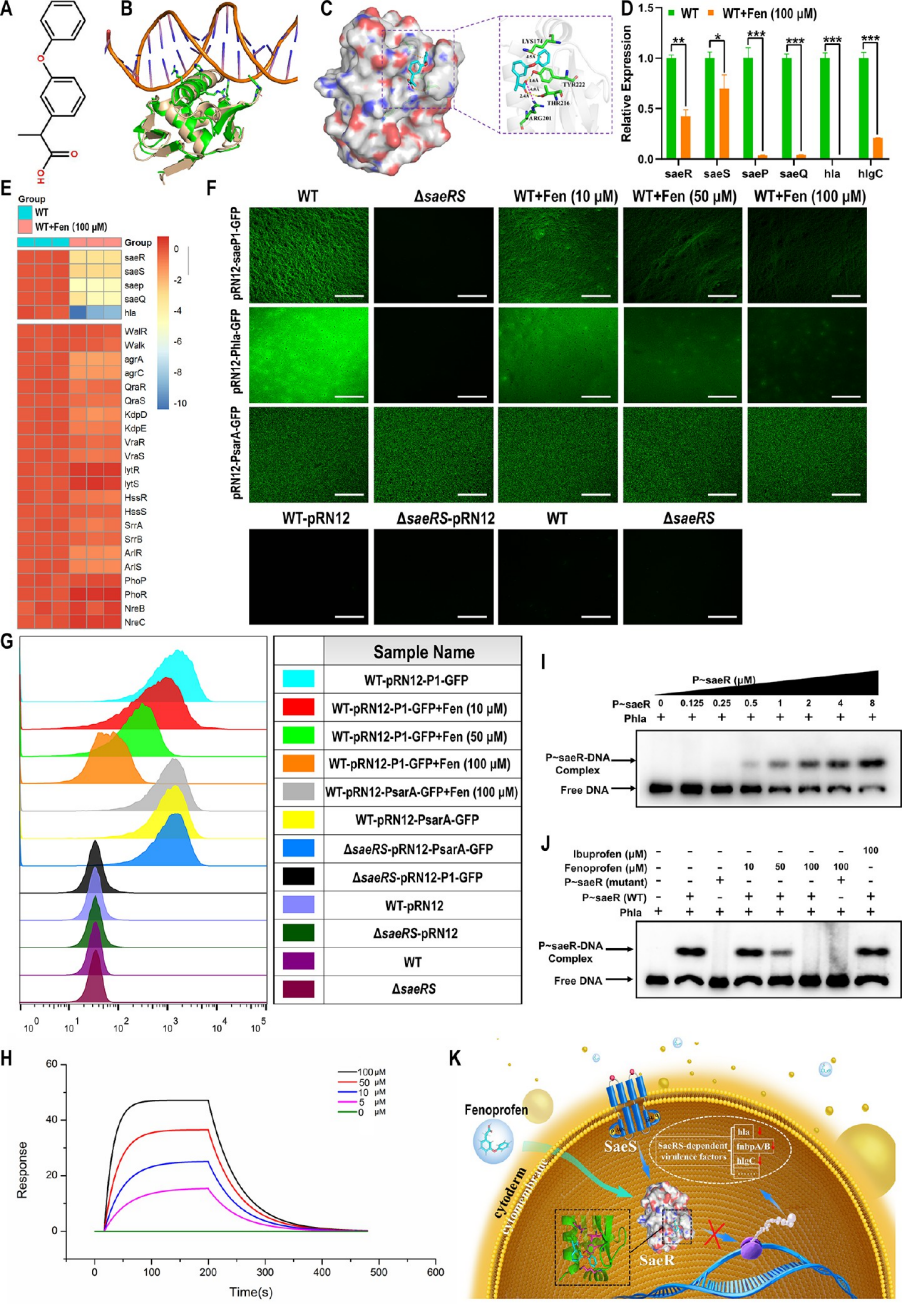
Fenoprofen was identified as an inhibitor of the SaeR protein. (A) Chemical structure of the fenoprofen agent. (B) Alignment of SaeR^DBD^ with the structure of PhoB-DNA (PDB ID: 1GXP). Coloring scheme: green, SaeR^DBD^; wheat, PhoB^DBD^; orange, DNA strands. The five key residues are shown as sticks. (C) Fenoprofen molecular docking result. The left panel shows the electrostatic potential surface of SaeR^DBD^, and fenoprofen is shown as cyan sticks. Residues interacting with fenoprofen are marked in green (right figure). Yellow, magenta, and orange dotted lines represent hydrogen bonds, salt bridges, and π–π stacking, respectively. (D) Inhibitory efficiency of fenoprofen on SaeRS TCS and *SaeRS*-dependent virulence factors. (E) Effect of fenoprofen on the two-component systems (TCSs) of *S. aureus* ST1792. The value represents log_2_ (fold change). (F) Fluorescence inhibition effect of different concentrations of fenoprofen on the GFP reporting system. Representative images were taken by CLSM. Scale bars = 200 μm. (G) Flow cytometry data of the effect of fenoprofen on the pRN12-P1-GFP reporting system. (H) Relative RU induced by fenoprofen in a dose-dependent model from 5 to 100 μM. (I,J) EMSA of SaeR with a biotin-labeled *hla* promoter fragment. The arrows indicate free DNA and the protein–DNA complex. (K) Schematic illustration of the antivirulence mechanism of fenoprofen. Fenoprofen had a high affinity for the SaeR protein and was able to prevent saeR binding to SBSs, resulting in a failure to activate transcription and expression of downstream virulence factors. All results are presented as the means ± SDs, and all experiments were performed in three biologically independent trials (D–J). *P < 0.05, **P < 0.01, and ***P < 0.001, and data were analyzed by two-tailed unpaired *t* tests (D).

The response regulator SaeR binds to the SaeR binding sequences (SBSs) of the downstream virulence gene promoter to activate transcription and is thus essential to the SaeRS TCS, making it an attractive and potential antivirulence drug target against *S. aureus* infection. To visualize the interacting surface between the DNA-binding domain of the response regulator SaeR (referred to as SaeR^DBD^, PDB ID: 4QWQ) and dsDNA, we homologously aligned the SaeR^DBD^ with the protein part of the PhoB-DNA complex (PDB ID: 1GXP)^35^ (Figure 2B). Some relevant studies have verified that five key residues (LYS174, HIS198, ARG199, ARG201, and TRP218) in the winged helix–turn–helix may interact with dsDNA,^36,37^ which was assumed to be a potential ligand binding site for compound screening. To identify inhibitors of the saeR protein that can bind to saeR^DBD^ and suppress the expression of virulence factors regulated by saeR, compounds from the Drug Bank database were used to perform structure-based virtual screening (SBVS). A molecular docking score was adopted to select the candidate molecular drugs. After inspecting the ligand and the target binding site interactions, SBVS resulted in the selection of 46 compounds (Figure S4). As NSAIDs are already widely used to relieve pain in the perioperative period after orthopedic surgery,^38−40^ we selected three NSAIDs (fenoprofen, oxaceprol, and naproxen) for the following study.

We constructed SaeRS GFP reporter strains based on *S. aureus* ST1792 (MSSA) and USA300 (MRSA) to test the efficacy of the drugs against the SaeR protein. It is well established that the *sae* operon contains four genes (*saeP*, *saeQ*, *saeR*, and *saeS*) and the two promoters P1 and P3.^13,41^ The stronger promoter, P1, is activated when the SaeR protein binds to its two SBSs. Promoter P1 was fused to a green fluorescence protein (GFP) segment in the plasmid pRN12 (Figure S5), and then the constructed plasmid (pRN12-P1-GFP) was inserted into *S. aureus* ST1792. Hence, if the promoter binding region of the SaeR protein was blocked by the agent and the protein could not bind to SBSs to activate promoter P1, then GFP expression was lower than that of the control group. Notably, fenoprofen was the most potent inhibitor, with a half-maximal inhibitory concentration (IC_50_) of 7.95 μM (Figure S6). As *hla* is one of the direct SaeRS TCS target genes and harbors SBS in its promoter region, we constructed a plasmid (pRN12-Phla-GFP) and tested the inhibitory efficacy of the three agents. In line with previous results, fenoprofen, with an IC_50_ value of 6.69 μM, was an effective SaeR protein inhibitor compared to oxaceprol and naproxen (Figure S6). Furthermore, similar results were obtained for MRSA USA300 (Figure S6), which is a major source of community-acquired infections in the USA.^42^ Compared to oxaceprol and naproxen, fenoprofen was a more potent inhibitor against the SaeR protein, so we chose it for the following study.

The docking score of fenoprofen was −6.403 kcal/mol, and it bound within the putative binding pocket in SaeR^DBD^ via the amino acids LYS174, ARG201, THR216 and TYR222 by π–π stacking, salt bridges or hydrogen bonds (Figure 2C). Specifically, fenoprofen is predicted to form hydrogen bonds with amino acid residues THR216 and TYR222 and salt bridges with ARG201, which provides electrostatic interactions. Moreover, π–π stacking interactions are predicted to occur between the drug and LYS174 residue, leading to a strong van der Waals interaction. Overall, these interactions drive the binding of fenoprofen with the protein, which produces a steric hindrance effect and thus prevents SaeR^DBD^ from interacting with dsDNA. Furthermore, the interactions between fenoprofen and two key residues (LYS174 and ARG201) may decrease the DNA-binding affinity of SaeR^DBD^. Therefore, fenoprofen may inhibit the SaeR protein and greatly affect the transcription process.

We treated *S. aureus* with fenoprofen (100 μM) for 24 h, and the qPCR results showed that the expression levels of the SaeRS TCS and downstream virulence factors were inhibited, but the expression levels of genes in other two-component systems were not affected (Figure 2D,E). To more intuitively observe the efficiency of fenoprofen in blocking the activation of the promoter, we cultured *S. aureus* ST1792 harboring the promoter-GFP-reporter plasmid with different concentrations of fenoprofen (10, 50, 100 μM) and then detected the fluorescence intensity of the treated *S. aureus* by CLSM. There are no SBSs on the promoter of sarA, so it does not bind to the saeR protein and is not activated by the saeR protein. We constructed pRN12-PsarA-GFP as a control GFP plasmid. Fenoprofen inhibited the transcriptional expression of GFP in a concentration-dependent manner (Figure 2F). This phenomenon was also demonstrated by flow cytometry, which showed that fenoprofen could inhibit GFP expression in *S. aureus* (Figure 2G and Figure S8). Taken together, these results demonstrated that fenoprofen inhibited the activation of promoter P1 and promoter *hla*.

### Fenoprofen Is Capable of Stably Blocking the Promoter Binding Region of the SaeR Protein

To verify whether fenoprofen is capable of binding to the functional domain of the SaeR protein, molecular dynamics (MD) simulations of fenoprofen and SaeR proteins were performed. The MD simulation data showed that fenoprofen bound to the promoter binding region of the SaeR protein and formed stable complexes with hydrogen bond interactions (Figure S9). Then, we overexpressed and purified the SaeR protein in vitro (Figure S10A,B). Surface plasmon resonance (SPR) titration and electrophoretic mobility shift assays (EMSAs) were used to test the binding affinity of fenoprofen to the SaeR protein. The relative SPR response unit (RU) was induced by fenoprofen in a dose-dependent manner from 5 to 100 μM, and the KD of fenoprofen was 16.5 μM (Figure 2H). The EMSA results showed that the SaeR protein displayed a specific affinity for the *hla* promoter fragment (Figure 2I and Figure S11C) and that the addition of fenoprofen could abolish this shift, but ibuprofen had no such effect (Figure 2J). We purified a recombinant saeR protein mutant lacking AG201, a critical amino acid residue in saeR protein that may interact with dsDNA (Figure S10C). In summary, these data indicated that fenoprofen had a high affinity for the SaeR protein and was able to prevent saeR binding to SBSs, resulting in a failure to activate transcription and expression of downstream virulence factors (Figure 2K).

### Fenoprofen Has Potent Antivirulence Efficiency by Inhibiting the Invasion and Biofilm Formation Abilities of *S*. *aure*us and Disrupting the Biofilm Structure

**Figure 3:**
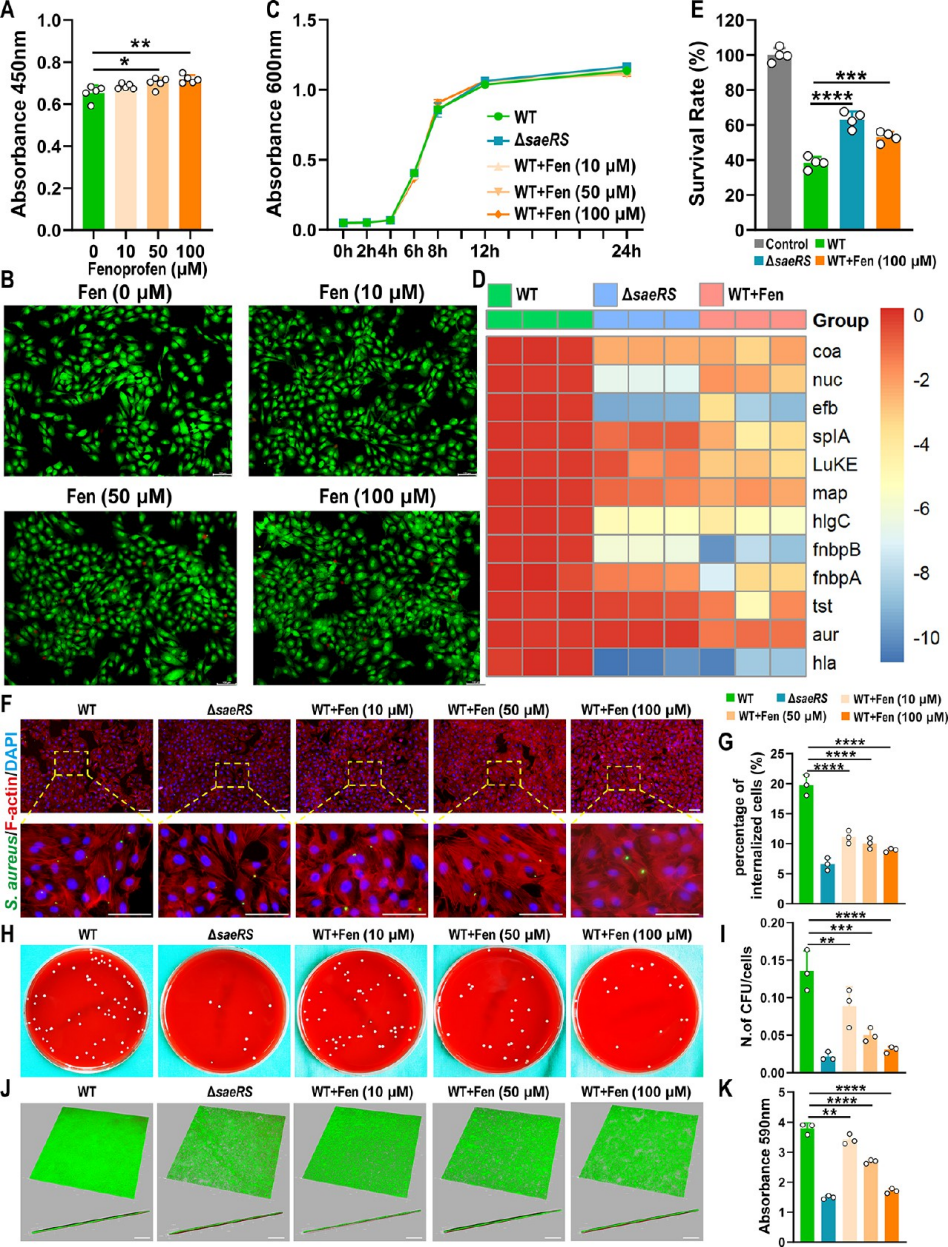
Antivirulence activity of fenoprofen and its effect on the invasion and biofilm formation abilities of *S. aureus*. (A) Effects of different concentrations of fenoprofen (0–100 μM) on the proliferation of MC3T3 cells (1 × 10^4^ cells/well) (*n* = 5). (B) Live/dead staining of MC3T3 cells treated with fenoprofen; green fluorescence represents living cells, and red fluorescence represents dead cells. (C) Effects of different concentrations of fenoprofen (0–100 μM) on bacterial growth. (D) Expression of *SaeRS*-dependent virulence genes was reduced after fenoprofen treatment. The value represents log_2_ (fold change). (E) Inhibitory effect of fenoprofen on the toxicity of *S. aureus*. Fenoprofen-treated or untreated bacterial suspensions were added to the MC3T3 cell culture medium (MOI = 20:1) and incubated for 6 h. CCK-8 was used to measure cell activity (*n* = 4). (F and G) Effects of different concentrations of fenoprofen (0–100 μM) on the internalization of *S. aureus* into MC3T3 cells. MC3T3 cells were stained with phalloidin (red) and DAPI (blue), and the number of *S. aureus* internalized into MC3T3 cells decreased with increasing fenoprofen concentrations (*n* = 3). (H and I) Spread plate results of the inhibitory effect of fenoprofen on the internalization ability of *S. aureus* and the corresponding number of *S. aureus* internalized into MC3T3 cells in each group (*n* = 3). (J) Effects of different concentrations of fenoprofen (0–100 μM) on *S. aureus* biofilm formation. Biofilms were stained with live/dead bacterial dyes, and 3D reconstruction of the biofilm was performed by CLSM. (K) Crystal violet staining was used to detect the antibiofilm ability of fenoprofen in 96-well tissue culture plates (*n* = 3). Scale bars, 100 μm (B and F) and 200 μm (J). All results are presented as the means ± SDs. *P < 0.05, **P < 0.01, ***P < 0.001, and ****P < 0.0001, and data were analyzed by one-way ANOVA (A, E, G, I, and K).

To explore whether fenoprofen was toxic to cells and whether it could be used in subsequent cell studies, we examined the cytotoxicity of fenoprofen on MC3T3 cells quantitatively and qualitatively. The CCK-8 results showed that fenoprofen did not exhibit cytotoxicity to MC3T3 cells at 100 μM (Figure 3A). Live/dead cell staining also showed that fenoprofen has excellent cell biocompatibility. Therefore, fenoprofen (less than 100 μM) did not cause a cell-killing effect (Figure 3B).

Fenoprofen can target and bind to the functional domain of the *S. aureus* SaeR protein, inhibiting *saeRS*-mediated activation of virulence gene expression. We analyzed the *saeRS*-mediated virulence gene expression of untreated and fenoprofen-treated WT strain by qPCR. The results showed that virulence gene expression was suppressed in the presence of 100 μM fenoprofen (Figure 3D). The overall virulence gene expression of the fenoprofen-treated WT strain was similar to that of the *saeRS* mutant strain, which indicated that fenoprofen could attenuate the pathogenicity of *S. aureus*. Next, we evaluated whether fenoprofen was capable of protecting cells from *S. aureus*-mediated cell injury. As expected, fenoprofen treatment improved the survival rate of cells infected with the WT strain ST1792 (Figure 3E). In agreement with these results, evident cell death (red fluorescence) was detected for the untreated WT strain-infected cells, but far fewer dead cells were observed in cells infected with the fenoprofen-treated WT strain or *saeRS* mutant strain (Figure S12A).

We noticed that the expression of *hla* and *hlgC* in fenoprofen-treated *S. aureus* ST1792 was reduced, so we used a hemolysis assay to verify this result. We found that fenoprofen treatment attenuated the hemolytic activity of the WT strain (Figure S12B). Furthermore, adding fenoprofen to staphylococcal cultures (up to 100 μM) did not hinder the growth of *S. aureus* ST1792 (Figure 3C). Hence, these results demonstrated that in vitro fenoprofen treatment attenuates *S. aureus* ST1792 virulence without affecting its growth.

Because *S. aureus* can evade host immune and antibiotic attacks by invading cells and forming biofilms on the surface of implants in orthopedic implant-associated infections, we evaluated the ability of untreated and fenoprofen-treated WT *S. aureus* ST1792 to invade cells and form biofilms. We observed that the internalization of *S. aureus* into MC3T3 cells was reduced after treatment with fenoprofen (Figure 3F,G). In addition, CFU counting results of intracellular *S. aureus* colonies confirmed that fenoprofen could limit the invasion of *S. aureus*, and the concentration of 100 μM resulted in the best effect (Figure 3H,I). Next, we determined the impact of fenoprofen on the biofilm formation of *S. aureus*. As presented in the CLSM images, the biofilm formed by the untreated WT strain appeared more compact, and the mean thickness values were higher than those values of the fenoprofen-treated WT strain and *saeRS* mutant strain (Figure 3J and Figure S12C). Interestingly, the biofilm structure formed by the fenoprofen-treated WT strain became loose and porous as the fenoprofen concentrations increased. Crystal violet staining showed that fenoprofen could inhibit *S. aureus* biofilm formation in a dose-dependent manner at concentrations of 10–100 μM (Figure 3K). Taken together, these findings support the claim that fenoprofen can limit the invasion and biofilm formation abilities of *S. aureus* and disrupt the structure of biofilms.

### Fenoprofen Inhibits the Adhesion of *S. aureus* to Implants and Reduces the Levels of eDNA and Protein in the Biofilm Matrix

**Figure 4:**
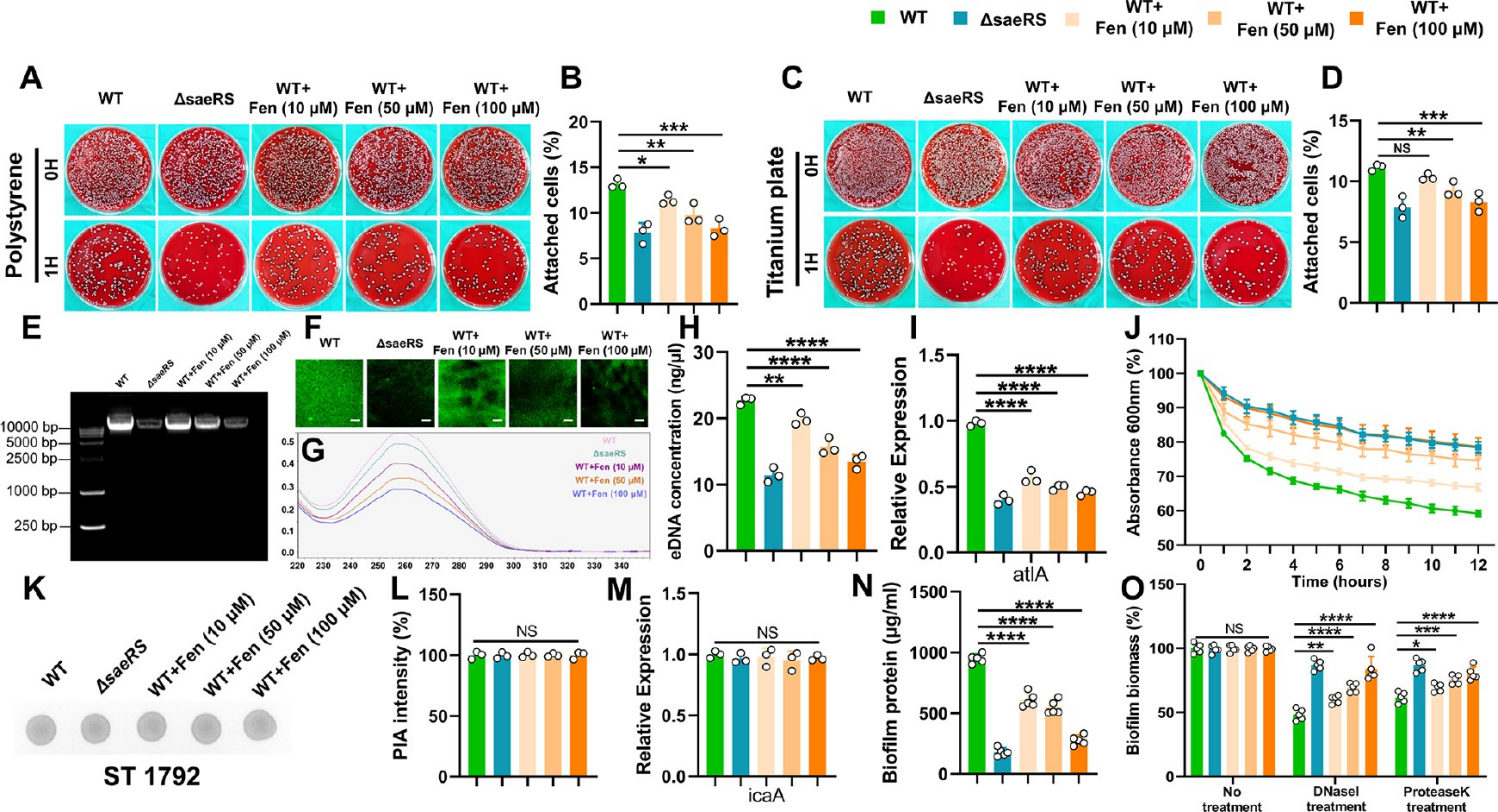
Effect of fenoprofen on the adhesion ability and biofilm matrix of *S. aureus* ST1792. (A–D) Spread plate results of the inhibitory effect of fenoprofen on the adhesion ability of *S. aureus* to polystyrene (A) and titanium plate (C) and the corresponding CFU counting results (B,D) (*n* = 3). (E) Agarose gel electrophoresis images of eDNA in the biofilms of each group. (F) SYTOX staining images of eDNA in the biofilms of each group. Scale bars, 200 μm. (G,H) Absorbance curve images and quantitative results of eDNA in the biofilms of each group (*n* = 3). (I) Effect of fenoprofen on the expression of atlA gene in *S. aureus* (*n* = 3). (J) Effect of fenoprofen on the Triton-X100 induced autolysis rate (*n* = 3). (K,L) Dot blot images and corresponding semiquantitative results of PIA in the biofilms of each group (*n* = 3). (M) Effect of fenoprofen on the expression of icaA gene in *S. aureus* (*n* = 3). (N) Quantitative results of protein in the biofilms of each group (*n* = 5). (O) Effect of DNase I and protease K on the biofilm formed by fenoprofen-treated and untreated *S. aureus* (*n* = 5). All results are presented as the means ± SDs. *P < 0.05, **P < 0.01, ***P < 0.001, and ****P < 0.0001, and data were analyzed by one-way ANOVA (B, D, H, I, L, M, N, and O).

In accordance with our previous qPCR results, fenoprofen treatment could reduce the expression of *fnbpA/B* in *S. aureus* ST1792 (Figure 3D). We first investigated the adhesion ability of *S. aureus* after feonoprofen treatment. The CFU results showed that fenoprofen treated *S. aureus* had poor adhesion to polystyrene and titanium surfaces (Figure 4A–D). Then we studied the effect of fenoprofen on the biofilm matrix. The extracellular DNA (eDNA) in the biofilms formed by fenoprofen treated and untreated *S. aureus* was extracted to quantitatively detect the effect of feonprofen on eDNA. We found that fenoprofen treatment reduced the eDNA content in biofilms (Figure 4E–H), which was also confirmed by fluorescent staining of eDNA in biofilms (Figure 4F). The bacteria in the biofilm will autolysis and then release eDNA. The expression of the autolysin-encoding gene atlA in fenoprofen treated *S. aureus* was downregulated compared with that in untreated *S. aureus* (Figure 4I). Meanwhile, lower Triton-X100-induced autolysis rates were observed in fenoprofen-treated *S. aureus* (Figure 4J). However, there was no significant difference in the content of PIA in each group of biofilms (Figure 4K,L). In addition, no statistical difference was observed in the transcriptional expression of icaA, which regulates the production of PIA (Figure 4M). Next, the protein contents in the biofilms formed by fenoprofen-treated and untreated *S. aureus* were measured to elucidate the effect of fenoprofen on the proteins in biofilms. The Quantitative results showed that fenoprofen could reduce the protein content in biofilms (Figure 4N). Furthermore, following treatment with DNase I and proteinase K, the biomass of the biofilms formed by fenoprofen-treated *S. aureus* decreased less compared to the untreated controls (Figure 4O), providing further evidence that fenoprofen inhibits biofilm formation and changes biofilms structure by affecting the content of eDNA and proteins in the biofilm matrix.

Moreover, we used the laboratory standard strain of *S. aureus*, ATCC 43300, to validate the antibiofilm effect of fenoprofen. The results showed that similar to the observations with ST1792, fenoprofen could inhibit the formation of biofilm by the 43300 strain in a concentration-dependent manner (Figure S13B**)**. Importantly, in the reconstructed images of biofilm staining, we observed that the biofilm formed by the fenoprofen-treated strain was thin, with a porous structure. In contrast, the biofilm formed by the untreated strain was thick and dense (Figure S13A,C). Subsequently, we revalidated the mechanism by which fenoprofen blocks biofilms in the ATCC 43300 strain. Similar to the conclusions obtained with ST1792, we observed that fenoprofen could inhibit the initial attachment of the 43300 strain to polystyrene and titanium surfaces, as well as decrease the content of eDNA and protein in the biofilm formed by the 43300 strain, but had no significant effect on PIA content (Figure S13D–R). In addition, we used RN4220, a chemically mutagenic defective strain derived from 8325-4 and formed PIA/PNAG type biofilms,^43,44^ to further verify the effect of fenoprofen on PIA. The results demonstrated that fenoprofen had no impact on the biofilm formation of RN4220 in TSB, TSBG, and TSB-NaCl (Figure S14A). Moreover, both proteinase K and DNase I also failed to degrade the biofilm formed by RN4220 (Figure S14B–D). This observation indicates that fenoprofen is unable to inhibit the formation of PIA/PNAG-type biofilm. Therefore, based on the experimental results obtained with the ST1792, 43300, and RN4220 strains, we concluded that fenoprofen inhibits the formation of the biofilm by affecting the initial attachment ability of strains during biofilm formation and by reducing the content of eDNA and protein in the biofilm matrix, while also changing the structure of the biofilm.

We also investigated the effect of fenoprofen on the biofilm-forming ability of clinical strain ST1792 and model strain ATCC 43300 in DEME, 20% synovial fluid, and 20% plasma culture conditions. The inhibition effect of fenoprofen on biofilms was still observed under different culture conditions (Figure S15A–F), indicating its broad applicability for the treatment of *S. aureus* biofilm infections. Besides, we evaluated the effect of fenoprofen on preformed biofilms, and the results showed that fenoprofen still had excellent antibiofilm efficacy against preformed biofilms in both ATCC 43300 and ST1792 strains (Figure S16A–C,F–H). The eDNA and protein contents in the fenoprofen-treated biofilm were less than those in the untreated group (Figure S16D,E,I,J), demonstrating that fenoprofen prevented the increase of matrix content in the biofilm, thereby preventing the biofilm from becoming more mature.

### Fenoprofen Treatment Suppresses Infection and Reduces Osteolysis in Orthopedic Implant-Associated *S. aureus* infection

**Figure 5:**
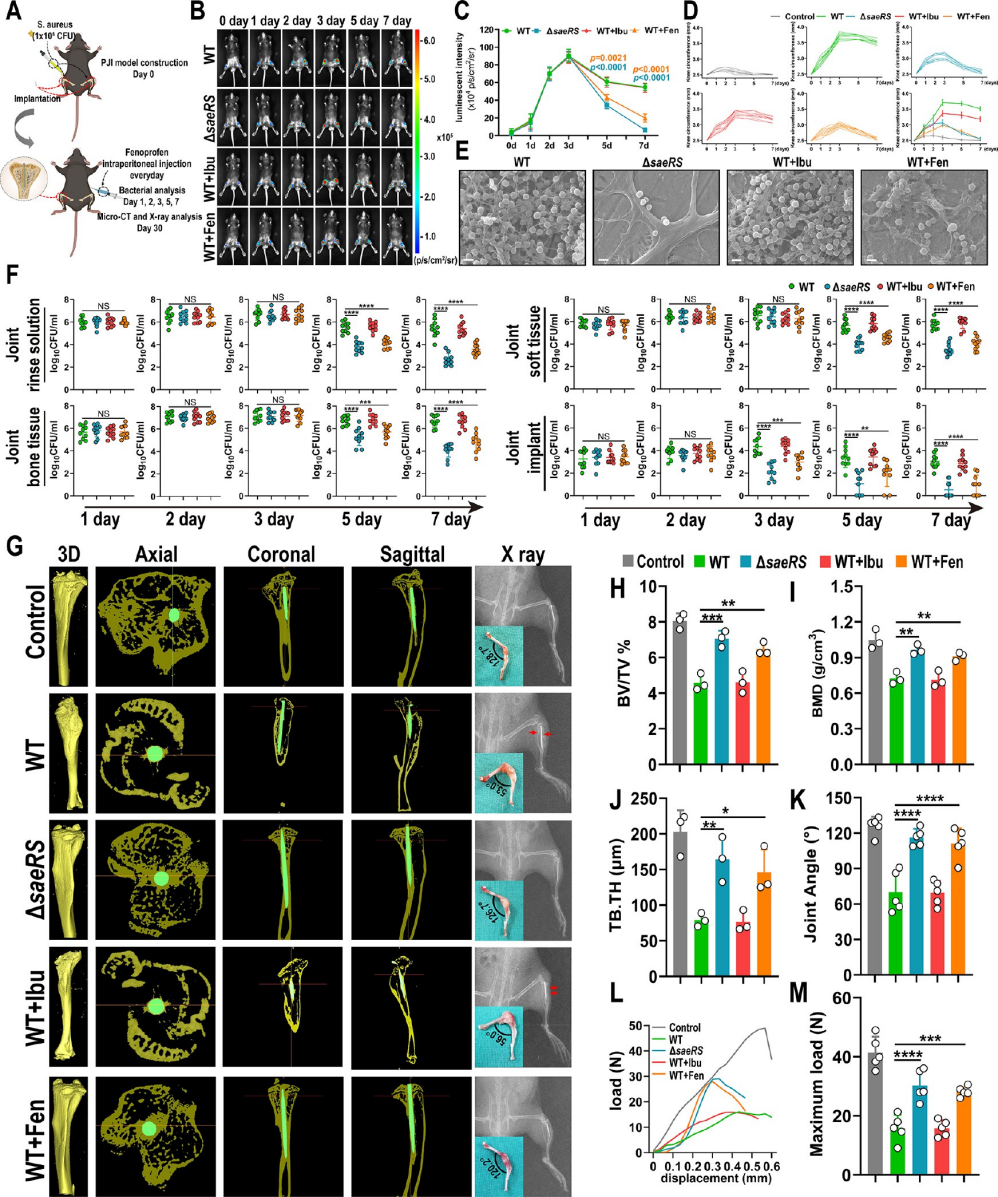
Effect of fenoprofen on orthopedic prosthetic joint infection. (A) Schematic diagram of prosthetic joint infection (PJI) model construction. Insulin needles were inserted into the tibia of mice as implants, and mouse articular cavities were injected with 1 × 10^6^ CFU *S. aureus*. Mice in the WT+Fen and WT+Ibu groups received intraperitoneal injections at a total dose of 100 mg/kg every day, and mice in the other groups were intraperitoneally injected with PBS. (B,C) Representative bioluminescence images and intensity in the PJI model at days 0, 1, 2, 3, 5, and 7. Color bar indicates the relative bacterial photon intensity (p/s/cm2/sr) (*n* = 3 for each time point). (D) Circumference of infected knee joints in PJI mice at days 0, 1, 2, 3, 5, and 7 (*n* = 10 for each time point). (E) SEM images of biofilm on the surface of knee prosthesis at day 7. Scale bars = 2 μm. (F) CFU counting results of the joint rinse solution, implants, peri-implant tissues and bone at days 1, 2, 3, 5, and 7 in the PJI model (*n* = 10; data are presented as individual points). (G) Axial, coronal, sagittal, and 3D reconstruction micro-CT images 4 weeks after infection. The mouse tibias are shown in yellow, and the implant is shown in green. X-ray and gross images of the mouse knee joint 4 weeks after infection. The red arrow indicates the osteolytic lesions and periosteal reaction. Control group (G to M): healthy mice that underwent tibia implant surgery but did not incur infection. (H–J) Quantitative data of micro-CT analysis including the cancellous bone mineral density (BMD), bone volume/total volume (BV/TV), and trabecular thickness (TB. TH) (*n* = 3; data are presented as individual points). (K) Quantitative data analysis of the mouse knee-bending angle 4 weeks after infection (*n* = 5; data are presented as individual points). (L,M) Mechanical properties of the tibia in mice 4 weeks after infection. Maximum vertical load (M) and corresponding mechanical curve (L) of tibia in each group (*n* = 5; data are presented as individual points). All results are presented as the means ± SDs. *P < 0.05, **P < 0.01, ***P < 0.001, and ****P < 0.001, and data were analyzed by two-way ANOVA with Tukey’s multiple comparison post-tests (C) and one-way ANOVA with Bonferroni multiple comparisons test (F, H, I, J, K, and M).

As fenoprofen attenuated the virulence and restricted the ability of *S. aureus* to evade the immune system in vitro, we next analyzed the in vivo efficacy of fenoprofen. Ibuprofen, another commonly used NSAID, was used as a control. We first confirmed that ibuprofen did not affect the growth of *S. aureus* and was not an inhibitor of the saeR protein (Figure S17). We generated a periprosthetic joint infection model, and mice received intraperitoneal injections of 100 mg/kg fenoprofen or ibuprofen every day (Figure 5A). Compared with the WT group, the bacterial bioluminescence intensity of the *saeRS* mutant group and WT+Fen group began to decrease at day 5 and day 7 (Figure 5B,C). Then, we measured the bacterial burden in the joint rinse solution, implants, peri-implant tissues, and bone at days 1, 2, 3, 5, and 7 after fenoprofen treatment. We noticed that the quantitative analysis of the bacterial burden in the implant showed a significant decrease in the fenoprofen treatment group and *saeRS* mutant infection group at day 3, while there was no significant difference among the 4 groups in the bacterial burden of the joint rinse solution, peri-implant tissues, and bone. At days 5 and 7, fenoprofen treatment decreased bacterial survival in all 4 samples compared with the untreated WT strain infection group (Figure 5F). In addition, the circumference of the infected knee decreased after fenoprofen treatment in PJI mice, indicating that the swelling of the knee gradually improved (Figure 5D). At day 7, the biofilm on the prosthesis removed from the knee joint of fenoprofen-treated mice was also disrupted, which is consistent with the bacterial count results (Figure 5E). Meanwhile, we did not find that treatment with ibuprofen, another commonly used clinical NSAID, would be helpful for the clearance of implant-associated infection (Figure 5B–F). This result indicated that the anti-infective effect of fenoporfen was attributed to its antivirulence efficiency rather than its anti-inflammatory and pain-relieving effects as NSAIDs. Together, these results demonstrate that fenoprofen treatment promotes the recovery of implant-associated *S. aureus* infection, which may be related to the suppression of implant biofilm formation and the disruption of biofilm structures by fenoprofen.

Orthopedic implant-associated infection can lead to bone resorption and periprosthetic osteolysis, and as a result, it is becoming one of the common causes of implant loosening.^45,46^ After determining that fenoprofen exhibited an excellent effect on implant-associated infection, we next sought to determine whether fenoprofen could inhibit infection-induced peri-implant osteolysis. We performed imaging assessments (X-ray and micro-CT) to evaluate the osteolysis of tibias. The micro-CT images demonstrated that, compared with the control group, severe osteolysis was observed around the implant with an enlarged intertrabecular space and decreased bone mass in the untreated WT strain infection group, whereas normal trabecular density and adequate bone mass were detected in the fenoprofen treatment group and *saeRS* mutant infection group (Figure 5G). Furthermore, the cortical bone mineral density (BMD), trabecular thickness (TB. TH), and peri-implant percent bone volume (bone volume/total volume, BV/TV) values increased in the fenoprofen treatment group and *saeRS* mutant infection group compared with that of the untreated WT strain infection group (Figure 5H–J). A similar trend was observed in the X-ray images, in which the degrees of bone destruction and osteolysis were apparently alleviated in the fenoprofen treatment group and *saeRS* mutant infection group (Figure 5G). Interestingly, we found that the joint bending angle in the untreated WT strain infection group was smaller than that in the control group, and the joint bending angles in the fenoprofen treatment group and *saeRS* mutant infection group were close to that in the control group, which means that fenoprofen can reduce the degree of implant-associated infection and prevent the development of purulent arthritis (Figure 5K). Furthermore, we measured the mechanical properties of the tibia in mice and showed that tibia in fenoprofen-treated infected mice were able to withstand heavier loads. The tibia in untreated infected mice, however, became brittle and more prone to fracture due to osteomyelitis and osteolysis caused by *S. aureus* infection (Figure 5L,M). Collectively, these in vivo data suggest that fenoprofen is effective in promoting the recovery of infection and inhibiting osteolysis in implant-associated *S. aureus* infections.

### Fenoprofen Treatment Can Restore the Walking Ability of Mice with Implant-Associated Osteomyelitis

In contrast to the treatment of other infections, the goal of orthopedic implant-associated infection treatment is not only to eradicate the infection but also to relieve pain and maintain joint function.^47,48^ Orthopedic implant-associated infection impairs the patient’s walking ability, which is caused by the pain and osteolysis resulting from the infection.^49^ Since fenoprofen is an NSAID with analgesic effects and can control implant-associated infection, we analyzed the effects of fenoprofen on the walking ability of mice with implant-associated osteomyelitis.

**Figure 6:**
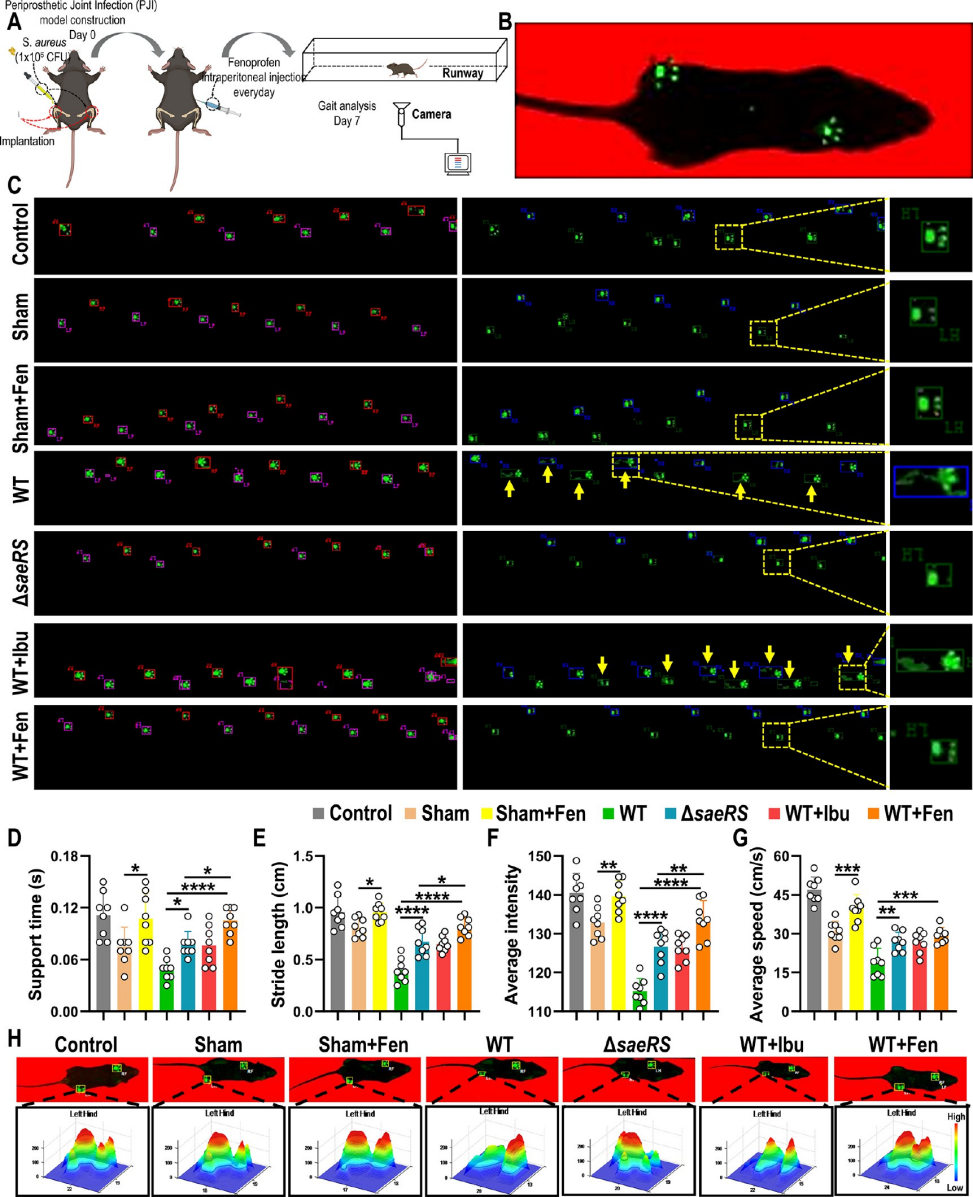
Fenoprofen helps restore the walking ability of mice with implant-associated osteomyelitis. (A) Schematic diagram of the gait experiment. To construct the PJI model, mice in the WT+Fen and WT+Ibu groups received intraperitoneal injections at a total dose of 100 mg/kg every day, and mice in the other groups were intraperitoneally injected with PBS. The gait test was performed on day 7. (B) Image of mice walking on the gait equipment tracks. (C) Actual trajectory of the mice as they walked and images of the mouse footprints. LF, left front; RF, right front; LH, left hind; and RH, right hind. Control: healthy mice that did not have surgery or infection. Sham: healthy mice that underwent tibia implant surgery but did not incur infection. Sham+fenoprofen: healthy mice that underwent tibia implant surgery but did not incur infection; the mice were treated with fenoprofen after surgery. WT: healthy mice that underwent tibia implant surgery; the mice were infected with *S. aureus* ST1792 (WT strain). Δ*saeRS*: healthy mice that underwent tibia implant surgery; the mice were infected with *S. aureus* ST1792-Δ*saeRS*. WT+Ibu: healthy mice that underwent tibia implant surgery; the mice were infected with *S. aureus* ST1792 and treated with ibuprofen after surgery. WT+Fen: healthy mice that underwent tibia implant surgery; the mice were infected with *S. aureus* ST1792 and treated with fenoprofen after surgery. Yellow arrows mark the shadow of the mouse footprint. (D–G) Quantitative analysis of the gait test data: support time, stride length, average intensity (average pressure on the runway), and average speed (*n* = 8; data are presented as individual points). (H) 3D reconstruction of left hind footprints of mice in each group. The color bar indicates the pressure intensity of each part of the mouse’s footprint on the ground. All results are presented as the means ± SDs. *P < 0.05, **P < 0.01, ***P < 0.001, and ****P < 0.001, and data were analyzed by one-way ANOVA with Bonferroni multiple comparisons test (D–G).

The gait of the mice was used to evaluate the effects of fenoprofen on the ability of the mice to walk (Figure 6A,B). In the untreated WT strain infection group, the footprints of the mice were straggled and irregular, indicating that the mice had limited walking ability due to osteomyelitis. In the fenoprofen-treated group and *saeRS* mutant infection group, the footprints of the mice were normal, well-defined and similar to those of the control group, demonstrating that fenoprofen could restore the posture and walking ability of osteomyelitis mice (Figure 6C). Furthermore, we measured the support time, stride length, average intensity, and average speed of each group. Compared to the sham group, the fenoprofen-treated sham group had better results concerning the support time, average intensity, and average speed, which demonstrated that the analgesic ability of fenoprofen was one of the reasons the walking abilities were restored. In addition, compared to the untreated WT strain infection group, the fenoprofen treatment group and *saeRS* mutant infection group both had better results for the four measurements, indicating that fenoprofen is effective in combating infection and can help mice regain their ability to walk (Figure 6D–G). 3D reconstruction images of the footprint showed that the footprints of the fenoprofen-treated sham group and WT strain infection group were similar to those of the control group, in which synergy could be observed between the soles and toes of mice as they walked. However, the footprint of the untreated WT strain infection group revealed that the mice relied on their toes to walk and did not place stress on the soles of their feet, so they were essentially walking on tiptoes (Figure 6H). Hence, these results indicate that fenoprofen treatment can restore the walking ability of implant-associated osteomyelitis mice, and this effect is attributed to the ability of fenoprofen to relieve pain and eliminate infection.

### Fenoprofen Treatment Impairs Biofilm Formation and Changes Biofilm Structure In Vivo

**Figure 7:**
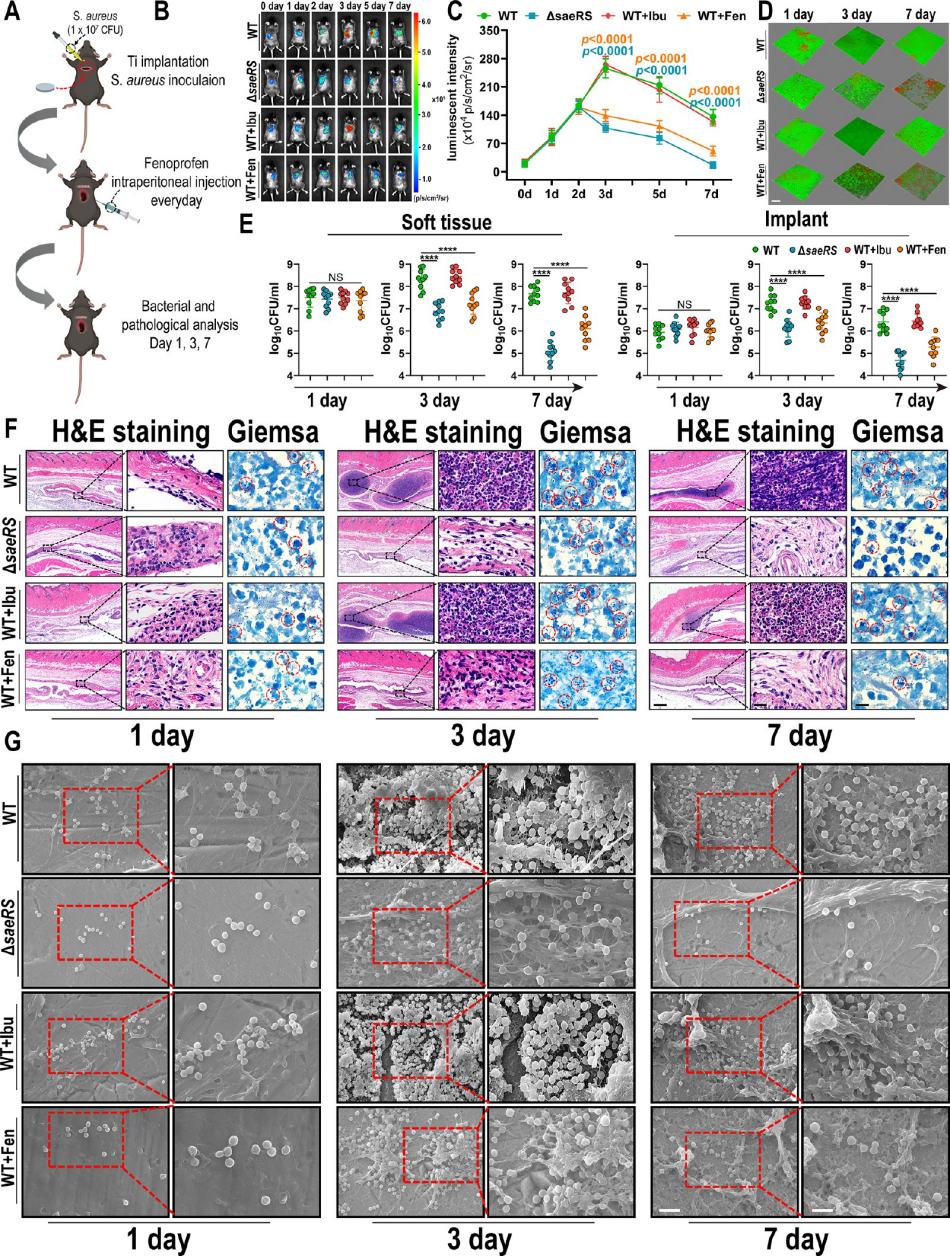
Antibiofilm efficiency of fenoprofen in vivo. (A) Schematic illustration of fenoprofen in vivo antibiofilm testing. Construction of the implant-associated biofilm infection model and the surface of the mouse implant were inoculated with 1 × 10^7^ CFU *S. aureus* ST1792-lux or ST1792-Δ*saeRS*-lux. Then, mice in the WT+Fen and WT+Ibu groups received intraperitoneal injections at a total dose of 100 mg/kg every day, and mice in the other groups were intraperitoneally injected with PBS. Bacteriological and pathological analyses were performed on days 1, 3, and 7. (B,C) Representative bioluminescence images and bioluminescence intensity of *S. aureus* implant infection on the backs of mice. The color bar indicates the relative bacterial photon intensity (p/s/cm2/sr) (*n* = 3 for each time point). (D) 3D confocal reconstruction of biofilms on the implant surface in mice at days 1, 3, and 7. Live bacteria were stained green (SYTO9), and dead bacteria were stained red (PI). (E) CFU counting results of the implant and surrounding infected soft tissue in the implant-associated *S. aureus* infection model at days 1, 3, and 7 (*n* = 10; data are presented as individual points). (F) H&E and Giemsa staining images of the implants surrounding the infected soft tissue at days 1, 3, and 7. Red circles indicate the bacterial residue in the soft tissue. (G) SEM images of the residual biofilms on the implant surfaces of various groups at days 1, 3, and 7. Scale bars = 5 and 2.5 μm, respectively. All results are presented as the means ± SDs. ****P < 0.001 and data were analyzed by two-way ANOVA with Tukey’s multiple comparison post-tests (C) and one-way ANOVA with Bonferroni multiple comparisons test (E).

Next, we wanted to explore the mechanism by which fenoprofen eliminates implant-associated *S. aureus* infection. As previous in vitro results showed that fenoprofen inhibited biofilm formation and disrupted the structure of *S. aureus* biofilms, we further validated the antibiofilm effect of fenoprofen in vivo using an implant-associated biofilm infection model (Figure 7A). During the observation period, the bioluminescence ascended from day 0 to day 3, peaked at day 3, and then began to decline in the WT and WT+Ibu groups. In comparison, after peaking at day 2, the average photointensity (infection severity) of the fenoprofen treatment infection group was lower than that of the untreated WT strain infection group from day 3 to day 7 (Figure 7B,C). To summarize, these data implied that fenoprofen could exert outstanding antibacterial activity against implant-associated biofilm infection in vivo.

Furthermore, to observe the structure of biofilms on implants after fenoprofen treatment in vivo, the implants (titanium plates) were collected and stained using live/dead fluorescent dyes at days 1, 3, and 7 after fenoprofen treatment. Immature biofilms were detected at day 1 on the surface of the implants in each group, indicating that *S. aureus* had begun to form biofilms. On day 3, confocal images showed that the implant was covered by live bacteria with a dense stacking biofilm structure in the untreated WT strain infection group, while loose and porous biofilms with few dead bacteria were observed in the fenoprofen treatment infection group and *saeRS* mutant infection group. At day 7, there were scattered dead bacteria and nearly no live bacteria in the biofilm of the fenoprofen treatment infection group and *saeRS* mutant infection group, while intact and dense biofilms could still be observed on the implant of the WT and WT+Ibu groups (Figure 7D). Similar trends and differences were confirmed by scanning electron microscopy (SEM) images (Figure 7G). These data indicated that fenoprofen could disrupt the structure of biofilms to make them easier for the host to eliminate.

To evaluate the histological changes and bacterial residuals of the peri-implant soft tissues in each group, the peri-implant soft tissues were harvested 1, 3, and 7 days after fenoprofen treatment and stained with H&E and Giemsa (Figure 7F). Severe inflammatory exudations, areas of necrosis, and neutrophil infiltrations were identified in the H&E-stained and immunofluorescence-stained slices in the WT and WT+Ibu groups, especially at day 3 and day 7. In contrast, we observed that the inflammatory reaction of the fenoprofen treatment infection group and *saeRS* mutant infection group was mild. Moreover, in the fenoprofen treatment infection group and *saeRS* mutant infection group, Giemsa staining indicated that the residual bacteria were decreased compared with that of the untreated WT strain infection group at day 3 and day 7. The CFU counting results of the implant and surrounding soft tissue also confirmed this finding (Figure 7E). These results verified that fenoprofen treatment was effective in eliminating implant-associated biofilm infections and further confirmed that this benefit was attributed to its antivirulence properties.

### Biofilms Formed by Fenoprofen-Treated *S. aureus* are Easier to Infiltrate and Eliminate by Leukocytes

**Figure 8:**
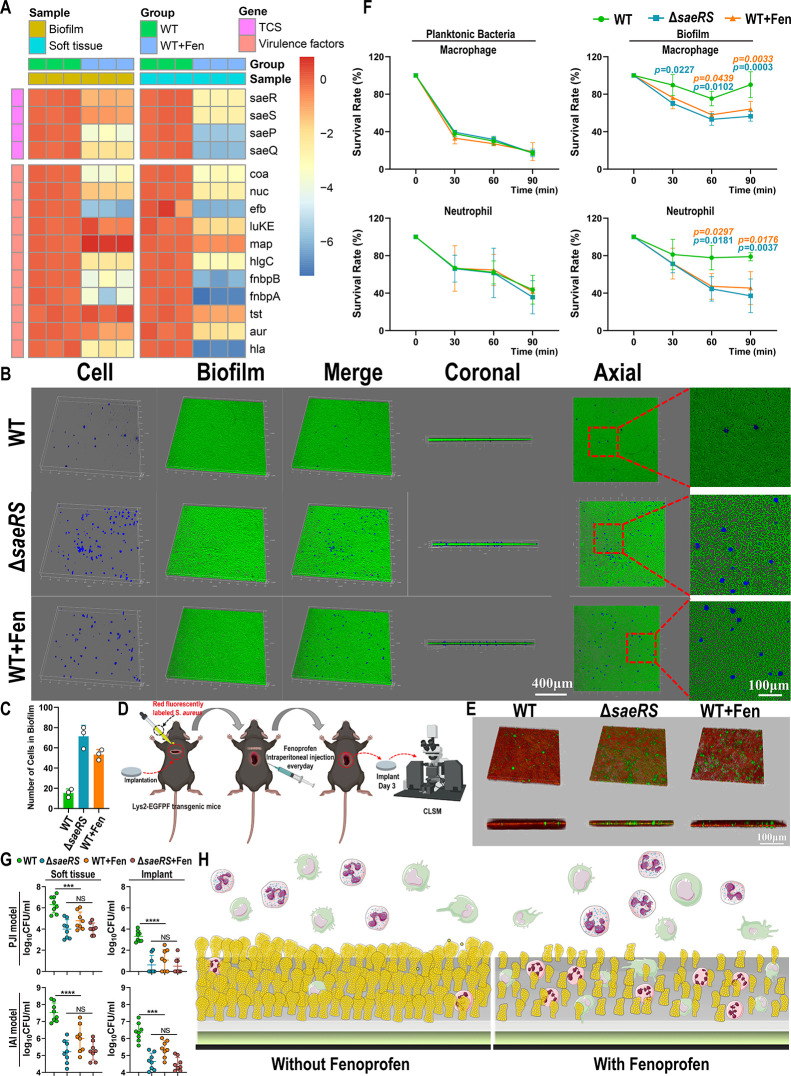
Fenoprofen induces *S. aureus* to form loose and porous biofilms that are more vulnerable to infiltration and elimination by leukocytes. (A) *saeRS*-dependent virulence gene expression levels in biofilms and soft tissue in an implant-associated infection mouse model 3 days after infection (*n* = 3). The value represents log_2_ (fold change). (B) Confocal laser scanning microscopy (CLSM) images of biofilms and macrophages. Biofilms were stained with SYTO9 (green) and PI (red), and the macrophages were stained with CellTrace Violet Cell Proliferation Dye (blue). Biofilms were grown for 24 h, and macrophages were added at equal numbers. (C) The number of macrophages infiltrating the biofilm was determined by CLSM images (*n* = 3; data are presented as individual points). (D) Schematic diagram of IAI model construction with Lys2-EGFPF transgenic mice and red fluorescence *S. aureus*. The surface of the mouse implant was inoculated with 1 × 10^7^ CFU *S. aureus* ST1792-pRN12-mCherry or ST1792-Δ*saeRS*-pRN12-mCherry. (E) CLSM images of biofilms in Lys2-EGFPF transgenic mice in vivo. The implant was removed on day 3 and washed with PBS, and then the biofilms and cells were photographed with CLSM. Myeloid-derived immune cells showed green fluorescence, while *S. aureus* showed red fluorescence. (F) Bactericidal rate of leukocytes against planktonic bacteria and biofilms in the WT, Δ*saeRS* and WT+Fen groups (*n* = 3). (G) Bacterial burden analysis of the implant and surrounding soft tissue in the PJI model and IAI model at day 7 (*n* = 8; data are presented as individual points). (H) Schematic diagram of the effect of fenoprofen on *S. aureus* implant-associated biofilm infection: fenoprofen treatment suppressed biofilm formation and changed the biofilm structure, which caused *S. aureus* to form loose and porous biofilms that were more vulnerable to infiltration and elimination by leukocytes. All results are presented as the means ± SDs. ***P < 0.001 and ****P < 0.001, and data were analyzed by two-way ANOVA with Tukey’s multiple comparison post-tests (F) and one-way ANOVA with Bonferroni multiple comparisons test (G).

The above results implied that the antibiofilm activity of fenoprofen might explain the in vivo antibacterial effect. Since we measured the inhibitory activity of fenoprofen on *saeRS*-dependent virulence gene expression in vitro, we identified whether fenoprofen inhibited the SaeR protein in vivo. Consistent with the in vitro results, fenoprofen reduced the expression of *saeRS*-dependent genes in vivo, verifying that fenoprofen could limit the function of the SaeR protein and suppress the virulence of *S. aureus* (Figure 8A).

The host innate immune system depends on leukocytes, mostly neutrophils and macrophages, to eliminate *S. aureus* infection. Previous studies have reported that intact and compact biofilms formed by *S. aureus* hinder the infiltration and phagocytosis of leukocytes, leading to “frustrated phagocytosis”.^22,50^ Encouraged by the antibiofilm activity of fenoprofen, which could modify the biofilm structure, we then set out to explore whether structural differences between the biofilms formed by fenoprofen-treated and untreated *S. aureus* influenced leukocyte infiltration. The amount of macrophages that infiltrated into biofilms formed by the fenoprofen-treated WT strain and *saeRS* mutant strain was much higher than that of untreated WT *S. aureus* biofilm, which was demonstrated using a live cell-specific stain (Figure 8B,C). In addition, when taking a closer look at the interactions of macrophages with biofilms, we observed that the biofilms formed by the fenoprofen-treated WT strain and *saeRS* mutant strain had loose structures, allowing macrophages to infiltrate and reside in the holes of the biofilms; in contrast, the untreated WT biofilm was intact and dense, making it difficult for macrophages to infiltrate into the biofilm. To verify this mechanism in vivo, a double fluorescence-labeled implant-associated biofilm infection was constructed using *S. aureus* with the mCherry plasmid and Lys2-EGFPF transgenic mice, which had fluorescence-positive myeloid-derived immune cells^51,52^ (Figure 8D and S18). Consequently, in line with the in vitro results, we found that more immune cells infiltrated the biofilms formed by the fenoprofen-treated WT strain and *saeRS* mutant strain based on the fluorescent images of myeloid-derived immune cell interactions with biofilms in vivo (Figure 8E). Taken together, these results demonstrated that the loose and porous biofilms formed by the fenoprofen-treated WT strain and *saeRS* mutant strain significantly increased the chances of leukocyte penetration.

Next, to confirm whether increased leukocyte penetration into biofilms improved leukocyte-mediated killing activity, we assayed the bactericidal activity of neutrophils and macrophages against planktonic *S. aureus* and biofilms. We observed that the biofilms formed by the fenoprofen-treated WT strain and *saeRS* mutant strain were more vulnerable to leukocyte-mediated killing compared to that of the untreated WT strain, whereas there was no apparent difference in the leukocyte-mediated killing activity against planktonic *S. aureus* (Figure 8F). To evaluate whether fenoprofen has an effect on host immunity, we constructed the PJI model and IAI model. CFU counting analysis of the implant and surrounding infected soft tissue was performed at 7 days after fenoprofen treatment. We did not observe a statistically significant difference in bacterial burden between the *saeRS* mutant group and the *saeRS* mutant strain with fenoprofen treatment group, suggesting that fenoprofen did not have a direct effect on host immunity (Figure 8G). These results indicated that differences in the structure of biofilms and subsequent differences in leukocyte-mediated killing ability were the reasons why fenoprofen could relieve implant-associated biofilm infection (Figure 8H).

### Antivirulence Effect of Fenoprofen Was Also Effective against Clinical Strains without Drug Resistance

To observe whether *S. aureus* developed resistance to fenoprofen, we exposed *S. aureus* to fenoprofen (100 μM) for 6 weeks and extracted RNA from the bacteria every day to assess the expression of *saeP* and *hla*. Meanwhile, we extracted DNA from the bacteria on day 7, day 14, day 21, day 28, day 35, and day 42, respectively, and performed PCR and sequencing of saeR to verify if spontaneous mutation occurred in the saeR sequence. The results showed that the expression of virulence genes regulated by *saeRS* were stably inhibited even after continuous exposure of ST1792 to fenoprofen for 6 weeks, and no spontaneous mutation of the saeR gene was observed (Figure S19A,B). In conclusion, it suggested that *S. aureus* did not develop drug resistance after continued fenoprofen treatment. To further confirm the clinical potential of fenoprofen against implant-associated infection, we examined the effect of fenoprofen on renal and liver function in mice. The data showed that fenoprofen treatment for 7 days had no significant effect on the renal and liver function of mice, and the internal organs of mice were morphologically normal (Figure S20).

Different strains of *S. aureus* can be isolated from patients with orthopedic implant-associated infections, including MSSA and MRSA, and each strain may have a different sensitivity to drugs. Hence, we investigated the antivirulence activity of fenoprofen against five MSSA strains and five MRSA strains that were isolated from patients with orthopedic implant-associated infection. First, growth curve data showed that fenoprofen did not affect the growth of all clinical strains (Figure S21). Since *hla* is a *saeRS*-dependent virulence gene and almost completely suppressed by fenoprofen in ST1792, the expression level of *hla* was utilized to evaluate the ability of fenoprofen to inhibit the SaeR protein in various strains. We exposed the *S. aureus* strains to fenoprofen (at a final concentration of 100 μM) for 24 h, and the qPCR results showed that fenoprofen had outstanding antivirulence activity against all clinical *S. aureus* strains (Figure S22A). Hemolysis tests also showed that fenoprofen could reduce the hemolysis ability of *S. aureus*, including both MSSA and MRSA (Figure S22B and Figure S23A). Next, we wanted to explore whether fenoprofen had an effect on the biofilms of different *S. aureus* strains. The data showed that fenoprofen could inhibit the biofilm formation of *S. aureus* strains (Figure S22C and Figure S23B). Notably, we still observed structural differences in the biofilms of fenoprofen-treated and untreated *S. aureus* clinical strains (Figure S22D). In summary, these findings revealed that fenoprofen could serve as an excellent inhibitor of SaeR function to combat implant-associated infection and suggested that fenoprofen has clinical application prospects.

## Discussion

As a major human pathogen, *S. aureus* causes a variety of infections, including orthopedic implant-associated infections (IAIs), which are a particular concern. The biofilm formation and internalization of *S. aureus* causes IAIs to become chronic infections,^53,54^ resulting in severe osteomyelitis and osteolysis. Here, structure-based virtual screening was used to discover the small-molecule agent fenoprofen, which is capable of selectively blocking the promoter binding region of the SaeR protein to inhibit SaeRS TCS function. The presence of fenoprofen leads to diminished binding of SaeR to the SaeR-binding sequence (SBSs), attenuating the expression of *saeRS*-dependent virulence. Of note, we demonstrated that fenoprofen eliminated orthopedic implant-associated infections through the following mechanisms: (i) for planktonic bacteria, fenoprofen depresses the ability of bacteria to invade nonprofessional phagocytes, and more planktonic bacteria are exposed to leukocyte attack as a result; and (ii) for adherent bacteria, fenoprofen causes bacteria to form loose and porous biofilms, making them more vulnerable to infiltration and elimination by leukocytes.

In *S. aureus*, the SaeRS TCS regulates the transcription and expression of many virulence factors,^55^ making it an ideal target for antivirulence agent development. Moreover, the high conservation of the saeR sequence in *S. aureus* demonstrates the significance of *saeRS* in regulating virulence, which ensures the effectiveness of saeR-targeting antivirulence drugs against most *S. aureus* strains. Unlike xanthoangelol B (1) and its derivative PM-56, which is an inhibitor of saeS,^27^ fenoprofen was screened and demonstrated to be an inhibitor of the SaeR protein. SaeR, an OmpR family response regulator and a transcription factor, binds to SBSs and activates target virulence gene transcription. Surface plasmon resonance (SPR) titration and electrophoretic mobility shift assays (EMSA) confirmed the binding affinity of fenoprofen to the SaeR protein, and MD simulations showed that fenoprofen can stably bind to the promoter binding region of the SaeR protein and maintain hydrogen bond interactions with the SaeR protein. The competitive inhibition of fenoprofen prevents the SaeR protein from performing its regulatory role as a transcription factor, and downstream virulence factors cannot be transcribed and expressed.

Nonsteroid anti-inflammatory drugs (NSAIDs) are recommended for pain relief during the perioperative period.^56^ Fenoprofen, which is a commonly used NSAID, reduces prostaglandin production by inhibiting cyclooxygenase (COX) activity.^57^ It is advised that the clinical dosage of fenoprofen should not exceed 3 g (UK) or 3.2 g (USA) per day;^58^ the concentration of the drug used in our experiment was below the clinical concentration, which means that fenoprofen may have new therapeutic potential at a reasonable concentration. This suggests that fenopeofen, as one of the adjuvant drugs perioperatively, may reduce the incidence of *S. aureus* biofilm infection. On the other hand, fenoprofen can assist antibiotic therapy after debridement of *S. aureus* implant infection. In recent years, NSAIDs have been reported to have anti-infective effects. Diflunisal was shown to attenuate skeletal cell death and bone destruction associated with *S. aureus* infection by inhibiting agrA and may be a potential treatment option for osteomyelitis; however, diflunisal may increase biofilm formation and is not suitable for treating implant-associated infection.^59,60^ Diclofenac, another widely used NSAID, was demonstrated to resensitize bacteria to β-lactams via the mecA/blaZ pathway,^61^ but it also has limitations: the synergistic effect of diclofenac and β-lactams is only applicable to MRSA, not to other antibiotic-resistant strains, and has not been validated in clinical strains. Here, we proposed and demonstrated a new mechanism of NSAIDs against infection through in vitro and in vivo experiments in which fenoprofen competitively attenuated the transcriptional regulatory function of the SaeR protein with potent antivirulence efficiency, and it was also validated in clinical MRSA and MSSA strains.

*S. aureus* forms biofilms that can act as physical and chemical barriers against antibiotics and immune system attacks, especially in implant-associated infections. The structure of a biofilm is critical to its function. Dense biofilms prevent leukocytes from infiltrating the biofilm; thus, leukocytes cannot kill bacteria in the biofilm.^62,63^ The biofilm formation process is regulated by many factors, such as *PSM*, *hla*, and *fnbpA/B*, and virulence factors play an important role.^64−66^ The effect of fenoprofen on *S. aureus* biofilm formation was observed both in vitro and in vivo; in particular, the biofilm structure was significantly changed. We demonstrated that compared to biofilms without fenoprofen treatment, the biofilms formed by fenoprofen-treated *S. aureus* were more easily infiltrated and eliminated by leukocytes. Notably, this phenomenon was detected in the Lys2-EGFPF transgenic implant-associated infection mouse model. Decreased expression of *fnbpA/B* in *S. aureus* weakened the ability of biofilms to attach to indwelling medical devices. In addition, many studies have found that inhibition of *hla* expression in *S. aureus* can weaken the formation of biofilms,^67,68^ but here, we further found that the structure of *S. aureus* biofilms became loose and porous at the same time, preventing bacteria from escaping immune system attack. Biofilms are sessile communities of bacteria embedded in an ECM that includes extracellular DNA (eDNA), polysaccharides, and proteins.^69^ eDNA contributes to biofilm formation and the stability of biofilm structures.^70,71^ We hypothesize that the change in *S. aureus* biofilm structure was related to its decreased adhesion capacity and the decreased expression of α-hemolysin in *S. aureus* after fenoprofen treatment, which inhibited bacterial and cell lysis and reduced eDNA during biofilm formation, resulting in loose and porous biofilms. However, more experiments are needed to verify this hypothesis.

*S. aureus* has been considered an extracellular bacterial pathogen for many years, but it has recently been accepted that *S. aureus* can invade various types of mammalian cells, such as endothelial cells^72,73^ and osteoblasts.^5,74^ The binding of fibronectin-binding protein A/B to α5β1 integrins on the cell surface enables *S. aureus* to adhere to host cells.^75^ Fenoprofen inhibited the transcription and expression of *fnbpA/B* and limited the ability of *S. aureus* to invade cells. Planktonic bacteria that are unable to escape host cells are more vulnerable to elimination by the immune system. Furthermore, we found that fenoprofen treatment also helped inhibit osteoporosis and osteomyelitis in implant-associated infection, which was consistent with a previous study stating that SaeRS TCS was critical for osteomyelitis pathogenesis.^29^

For clinical orthopedic surgeons, restoring the patient’s ability to walk is the treatment goal. Patients with implant-associated *S. aureus* infection are often unable to walk due to pain, which can lead to joint stiffness and other severe complications. We demonstrated that fenoprofen had a positive effect on restoring the ability to walk. This is attributed to the combined effects of the anti-inflammatory and pain-relieving properties of fenoprofen and its ability to reduce the virulence of *S. aureus*, thereby helping to eliminate *S. aureus* infection.

The goal of our study was to find effective drugs for the treatment of implant-associated *S. aureus* infections in the clinic. We demonstrated that fenoprofen had excellent antivirulence effects against all clinical strains, including both MSSA and MRSA. This may be attributed to the conservation of the SaeRS TCS in *S. aureus*. Moreover, we showed that *S. aureus* did not develop drug resistance. The potential explanation may be that, on the one hand, the drug does not exert pressure on bacterial growth; on the other hand, the transcriptional regulation function of SaeR on downstream virulence genes cannot be replaced or compensated by other TCSs. We envision fenoprofen as an anti-inflammatory analgesic and infection prevention agent after orthopedic surgery and as an adjuvant compound for combinatorial usage in anti-infective therapy.

There are some limitations to our study. The biofilm formation and invasion of *S. aureus* are regulated by many factors, and the mechanism by which fenoprofen attenuates *S. aureus* immune escape still needs further analysis. Whether fenoprofen has an effect on the host immune system also remains unclear. In addition, we need to perform further chemical modifications and design drug delivery systems to improve the drug efficiency.

In summary, this work identified fenoprofen as a potent antivirulence agent with potential clinical application value, as it competitively binds to the SaeR protein, suggesting that SaeR is an attractive and druggable target against implant-associated *S. aureus* infection. This study also provides ideas for developing new nonantibiotic drugs for the treatment of implant-associated *S. aureus* infection.

## Materials and Methods

### Bacterial Strains, Plasmids, And Growth Conditions

The bacterial strains and plasmids used in this paper were listed in Table 1 of the Supporting Information. Clinical isolates of methicillin-sensitive *Staphylococcus aureus* (MSSA) (strain ST1792, strain 73547, strain 77939, strain 82237, strain 73156, and strain 73574) and methicillin-resistant *Staphylococcus aureus* (MRSA) (strain 84822, strain 82300, strain 85116, strain 82855, and strain 81682) were obtained from the Shanghai Jiao Tong University Affiliated Sixth People’s Hospital. *S. aureus* USA300 was kindly provided by Dr B. Diep, and *saeRS* mutants in the *S. aureus* ST1792 were constructed by our laboratory. Luminescent strain ST1792-LUX was constructed and preserved in the laboratory and was used for real-time monitoring implant-associated infection via bioluminescence imaging. The construction of fluorescence-labeled *S. aureus* has been described previously,^76^ and the strains were used for fluorescence imaging. ATCC 43300 was derived from a laboratory frozen stock culture. All strains were stored at −80 °C before use. Prior to each experiment, the bacteria was inoculated into 4 mL tryptic soy broth (TSB, Haibo, Qingdao, China) or TSBg (TSB with 0.5% glucose, for biofilm experiments) and incubated overnight at 37 °C and diluted to the corresponding concentration according to experimental requirements.

### Chemicals

Fenoprofen calcium dihydrate (Top Science, Shanghai, China, 99.29% purity), Naproxen (Top Science, Shanghai, China, 99.91% purity), Oxaceprol (Top Science, Shanghai, China, 98.00% purity), and Ibuprofen (Top Science, Shanghai, China, 99.7% purity) were used. The drugs were dissolved in DMSO (dimethyl sulfoxide) into 100 mM and then diluted to the experimental concentration of the response.

### Animal Experiments

All animal experiments and operations in this paper were approved by the Animal Care and Experiment Committee of Shanghai Jiao Tong University Affiliated Sixth People’s Hospital and in compliance with the NIH Guide for Care and Use of Laboratory Animals guidelines. The construction methods of Periprosthetic Joint Infection (PJI) model and Implant-associated Biofilm Infection Model are shown in the Supporting Information.

### Statistical Analyses

All statistical analyses in this study were performed using GraphPad Prism version 8.4.3. Two-tailed Student’s *t* test was used to analyze the statistical difference between the two groups, and one-way or two-way analysis of variance (ANOVA) was used to analyze the statistical difference between the multiple groups after passing the normal distribution test. Shapiro-Wilk test was used to verify the normality of the data. *P* < 0.05 was considered statistically significant. Error bars show the mean ± SD.

## Data Availability

All data associated with this study are present in the paper or the Supporting Information.

## Supporting Information Available

Materials and methods; (Figure S1) schematic diagram of fenoprofen for the treatment of orthopedic implant-associated infection; (Figure S2) virulence expression and hemolytic ability of *saeRS* mutant strains were decreased; (Figure S3) schematic of mouse implant-associated biofilm infection model construction; (Figure S4) procedure for virtual screening; (Figure S5) schematic diagram of P1/Phla-GFP reporter plasmid construction; (Figure S6) efficiency of fenoprofen in inhibiting transcriptional activation of SaeR protein; (Figure S7) saeR sequence of *Staphylococcus aureus* is highly conserved; (Figure S8) Fenoprofen attenuates the ability of SaeR protein to activate promoters; (Figure S9) molecular dynamics (MD) simulation results of SaeR-fenoprofen complex; (Figure S10) expression and purification of SaeR protein; (Figure S11) saeR protein needs to be phosphorylated to bind to the *hla* promoter and this binding is specific; (Figure S12) effect of fenoprofen on toxicity, hemolysis, and biofilm formation of *S. aureus*; (Figure S13) effect of fenoprofen on the adhesion ability, biofilm formation, and biofilm matrix of *S. aureus* ATCC 43300; (Figure S14) antibiofilm activity of fenoprofen against *S. aureus* RN4220 strain; (Figure S15) antibiofilm effect of fenoprofen on *S. aureus* ATCC 43300 and ST1792 under different culture conditions; (Figure S16) fenoprofen prevents preformed biofilms from maturing; (Figure S17) Ibuprofen is not an inhibitor of *S. aureus* saeR protein; (Figure S18) construction and identification of Lys2-eGFPF mice; (Figure S19) *S. aureus* does not develop drug resistance to fenoprofen; (Figure S20) anti-infective dose of fenoprofen does not cause toxicity to mice and has excellent biocompatibility in vivo; (Figure S21) effects of fenoprofen on the growth ability of clinical strains; (Figure S22) fenoprofen has excellent efficacy against clinical strains of *S. aureus*; (Figure S23) fenoprofen attenuated the hemolysis and biofilm formation abilities of *S. aureus* clinical strains; (Table S1) plasmids and strains used in this study; (Table S2) primers used in this study (PDF)
The ranking, structures, and docking scores of the top 46 compounds obtained through virtual screening (XLSX)
