# Supplementary material for: Repurposed Fenoprofen Targeting SaeR Attenuates Staphylococcus aureus Virulence in Implant-Associated Infections
Source: ACS Cent Sci. 2023 Jun 15;9(7):1354–73. doi: 10.1021/acscentsci.3c00499 (PMC10375895; doi:10.1021/acscentsci.3c00499)
Supplement: Supplementary file 1 — oc3c00499_si_001.pdf [file oc3c00499_si_001.pdf]

Supplementary Materials for

Repurposed fenoprofen targeting SaeR attenuates Staphylococcus aureus virulence in implant-associated infections

Feng Jiang[1,†]; Yingjia Chen[2,3,†]; Jinlong Yu[1,†]; Feiyang Zhang[1]; Qian Liu[4]; Lei He[4]; Musha Hamushan[1]; Jiafei Du[1]; Boyong Wang[1]; Pei Han[1]; Xiaohua Chen[7]; Jin Tang[5,*]; Min Li[4,6,*]; Hao Shen[1, *]

Supplementary Results

The PDF file includes:

Materials and Methods

Fig. S1. Schematic diagram of fenoprofen for the treatment of orthopedic implant-associated infection.

Fig. S2. The virulence expression and hemolytic ability of *saeRS* mutant strains were decreased.

Fig. S3. Schematic of mouse implant-associated biofilm infection model construction.

Fig. S4. Procedure for virtual screening.

Fig. S5. Schematic diagram of P1/Phla-GFP reporter plasmid construction.

Fig. S6. Efficiency of fenoprofen in inhibiting transcriptional activation of SaeR protein.

Fig. S7. The saeR sequence of Staphylococcus aureus is highly conserved.

Fig. S8. Fenoprofen attenuates the ability of SaeR protein to activate promoters.

Fig. S9. Molecular dynamics (MD) simulation results of SaeR-fenoprofen complex.

Fig. S10. Expression and purification of SaeR protein.

Fig. S11. The saeR protein needs to be phosphorylated to bind to the *hla* promoter and this binding is specific.

Fig. S12. Effect of fenoprofen on toxicity, hemolysis and biofilm formation of *S. aureus*.

Fig. S13. The effect of fenoprofen on the adhesion ability, biofilm formation, and biofilm matrix of *S. aureus* ATCC 43300.

Fig. S14. Anti-biofilm activity of fenoprofen against *S. aureus* RN4220 strain.

Fig. S15. The anti-biofilm effect of fenoprofen on *S. aureus* ATCC 43300 and ST1792 under different culture conditions.

Fig. S16. Fenoprofen prevents pre-formed biofilms from maturing.

Fig. S17. Ibuprofen is not an inhibitor of *S. aureus* saeR protein.

Fig. S18. Construction and identification of Lys2-eGFPF mice.

Fig. S19. *S. aureus* does not develop drug resistance to fenoprofen.

Fig. S20. The anti-infective dose of fenoprofen does not cause toxicity to mice and has excellent biocompatibility in vivo.

Fig. S21. Effects of fenoprofen on the growth ability of clinical strains.

Fig. S22. Fenoprofen has excellent efficacy against clinical strains of *S. aureus*.

Fig. S23. Fenoprofen attenuated the hemolysis and biofilm formation abilities of *S. aureus* clinical strains.

Table S1. Plasmids and strains used in this study.

Table S2. Primers used in this study.

Materials and Methods

***S. aureus* RNA extraction and real-time quantitative PCR.** To detect the difference in virulence factors expression in ST1792 and its isogenic *saeRS* mutant, bacteria were cultured in TSB in a shaking incubator for 24 h at 37 ºC, 200 rpm. To test the inhibitory efficiency of fenoprofen on *saeRS*-dependent virulence factors expression in vitro, *S. aureus* ST1792 was co-cultured with the drug (100 μM) in TSB for 24 h at 37°C, 200 rpm. To test the inhibitory efficiency of fenoprofen on *saeRS*-dependent virulence factors expression in vivo, bacteria were collected from the soft tissue of the abscess and from the biofilms on the implant surface on the third day after infection. The bacteria mentioned above were harvested and transferred to a tissue lyser (ScientzTM, Ningbo, China), then the cell membrane of bacteria was physically disrupted for 40 s of 70 hz, repeat three times. The total RNA was extracted with Ezscript Reverse Transcription Kit (EZBiosciebce, USA) following the manufacturer's instructions and then was reverse transcribed into cDNA using the SYBR PrimerScript RT-PCR kit (EZBiosciebce, USA). The synthesized cDNA was stored at −80 °C. The expression levels for the target gene in each sample were determined by a real-time quantitative PCR according to the following protocol: 5 min at 95 °C, 1 cycle; 10 s at 95 °C and 30 s at 60 °C, 40 cycles. The relative primers were listed in **Supplementary Table 2**. The $2^{-\Delta\Delta Ct}$ method was used to calculate the relative RNA level of each gene, using gyrb as reference gene. Each reaction was performed three times.

Hemolytic ring test. *S. aureus* ST1792 and its isogenic *saeRS* mutant were cultured in TSB for 24 h at 37 ºC. 2 μl bacterial suspension was dropped onto the sheep blood agar plate and dried for 5 min at room temperature. Then the agar plate was cultured at 37 °C for 24 h and taken for photographs.

Structure-based virtual screening. The three-dimensional structures of 8823 small molecule drugs were downloaded from Drug Bank Small Molecule database (the version of 5.1.7) which was released on July 02, 2020. Lipinski's rule-of-five (molecular weight ≤ 500, number of hydrogen-bond donors ≤ 5, number of hydrogen-bond acceptors ≤ 10, and log P ≤ 5.0)[1] were applied to process the original drug set. Then, Pan Assay Interference Compounds (PAINS) filters[2] were employed to remove several promiscuous bioactive compounds. The above two processes were conducted with the RDKit library and the final drug set consisting of 6536 compounds was used as the ligand database.

Firstly, the 6536 compounds were prepared using LigPrep (version 3.4; Schrödinger, LLC: New York, NY, 2015), which was able to generate stereoisomers and tautomers. The small molecules were protonated at pH 7.4 ± 0.0 with Epik[3]. Secondly, The crystal structure of DNA-binding domain of the response regulator SaeR (referred to as $SaeR^{DBD}$, PDB access code: 4QWQ) was downloaded from the Protein Data Bank[4] and $SaeR^{DBD}$ was homologous aligned with the protein portion of the PhoB-DNA complex using software. Thirdly, the protein structure was prepared with the Protein Preparation Wizard Module provided in the Maestro program of Schrödinger software (Schrödinger, LLC: New York, NY, 2015). The protein structure was prepared with the following workflow: assigning bond orders, adding hydrogens, creating zero-order bonds to metals, creating disulfide bonds, deleting water molecules >3 Å from the het group, removing waters with less than three H-bonds to non-waters, and restraining the minimization to allow only hydrogen atoms to be freely minimized. Next, based on the optimized protein structures, the Receptor Grid Generation Module

of Schrödinger software was used to generate the grid for docking and the grid files were defined as a 10 × 10 × 10 Å region centered at the selected residues(LYS174, ARG201 and TRP218) of the protein[4]. Finally, the prepared small molecules with different low energy configurations were docked to the corresponding target protein grid files by Glide program (Schrödinger, LLC: New York, NY, 2015), with the SP precision mode and the default parameters[5].

Construction of GFP reporter strains (GFP-promoter fusion). Sae P1 promoter fragment and *hla* promoter fragment were obtained from the DNA of ST1792 by PCR. Then we obtained pRN12-GFP fragment from pRN12 plasmid[6] by PCR. To construct the GFP reporter strain, Hieff Clone® Plus Multi One Step Cloning Kit (Yeasen, Shanghai, China) was used to link promoter fragments to plasmid fragments and finally constructed pRN12-P1-GFP plasmid and pRN12-Phla-GFP plasmid. Next, the plasmids were transformed into *E. coli* DH5α and were verified by PCR sequencing. The plasmids that has been verified were transformed into *S. aureus* competent cells RN4220 via electroporation and maintained using chloramphenicol (10 μg/ml). Finally, the plasmids were transduced into *S. aureus* ST1792 (MSSA) and USA300 (MRSA) with ϕ11.

Half-maximal Inhibitory Concentration (IC50) Analysis. GFP reporter strains were treated with different concentrations of drugs (fenoprofen, naproxen, oxaceprol) for 24 h, the control group was treated with the same concentration of DMSO. Then, fluorescence signal intensity of each group were measured by the microplate reader (BIO-TEK, ELX 800) at 485 nm excitation/530 nm emission. The number of bacteria was calculated by spread-plate method (SPM)[7]. Briefly, the bacterial solution was serially diluted 10-fold with sterile PBS, and then 100 μl of each diluted bacterial solution was spread on the blood plate and incubated overnight at 37℃. The number of bacteria in the solution was calculated from the number of colonies on the blood plate. Average fluorescence intensity of single *S. aureus* = Total fluorescence intensity/ bacteria number. Percentage of fluorescence intensity = (fluorescence intensity of single *S. aureus* in the sample) / (fluorescence intensity of single *S. aureus* in the control group)*100%.

Confocal laser scanning microscopy (CLSM) of the GFP reporter strains. Briefly, GFP reporter strains (ST1792:: pRN12-P1-GFP and ST1792:: pRN12-Phla-GFP) were treated with fenoprofen (10, 50, 100 μM) in TSB at 37 ºC for 24 h and the other strains were treated with the same concentration of DMSO (0.1%). After 24 h, the bacterial suspension of each groups were adjusted to OD_{600}=0.8 with TSB. Next, 100 μl bacterial suspension was added to the 96-well plate and centrifuged at 6000 rpm for 5 min. Lastly, fluorescence of the GFP reporter strains were observed by a confocal microscope (Leica TCS SP8, Germany).

Flow cytometry test. As described above, GFP reporter strains were treated with fenoprofen in different concentrations in TSB at 37 ºC for 24 h. Then the bacteria were washed twice and resuspended in fresh PBS to make bacterial suspensions with an OD600 of 0.8. Fluorescence intensity of the strains were measured in a Fortessa flow cytometer (Beckton Dickinson, USA) in FITC channel.

Protein expression and purification. The primers in this study were designed according to the sequence of *S. aureus* MW2 genome (NC-003923) published on NCBI. The full-length fragment of

SaeR gene was amplified using the genomic DNA of ST1792 strain as template. After BamH I and XhoI double restriction digestion, the SaeR fragment was inserted into the pET28a plasmid and then transferred into *E.coli* DH5α. The recombinant plasmid PET28a-saeR was identified by PCR and DNA sequencing and then transferred into the expression strain *E.coli* BL21 (DE3) to obtain the recombinant expression strain BL21-PET28a-saeR. The recombinant strain BL21-PET28a-saeR was inoculated with 1000 ml Kan^+ (50 μg/mL) LB liquid at the ratio of 1:200 and cultured to OD_{600} ≈ 0.6. IPTG (Shengong, shanghai) was added to the final concentration of 0.4 mmol/L and induced at 25 °C for 12 h. The bacteria were collected by centrifugation and washed with PBS for 2 times. The bacteria were suspended with 25 ml non-denatured binding buffer (20 mmol/L imidazole, 500 mmol/L NaCl, 20 mmol/L Tris, 10% Glycerol, pH 8.0) and ultrasonic cracking in ice (Qsonica ,USA, power 200 W, ultrasonic 5 s, interval 10 s, a total of 2-3 h), centrifugation at 4 °C, 15 000 g for 1 h. The supernatant was collected and added to the balanced Ni-NTA Agarose affinity chromatography column. 30 ml non-denaturing rinse buffer (250 mmol/L imidazole, 500mmol/L NaCl, 20 mmol/L Tris, 10% glycerol, pH 8.0) was added to elute target protein. SDS-PAGE analysis of recombinant proteins. The SaeR protein was stored at -80°C.

Surface Plasmon Resonance (SPR) analysis. SPR experiments were performed using a OpenSPRTM system (Nicoya, Canada). First, install the NTA chip according to OpenSPRTM instrument standard operating procedure and run at maximum flow rate (150 μl/min) to test buffer PBS (PH 7.4) . After reaching the signal baseline, adjust the flow rate of the buffer to 20 μl /min. The prepared imidazole and $NiCl_2$ solutions were taken out and injected through the injection port to complete the chip surface functionalization. The SaeR protein was diluted with PBS (PH 7.4) and 200 μl protein suspension was injected through the injection port to interact for 4 min. Baseline was observed for 5 min to ensure stability. Fenoprofen was prepared at concentrations of 5, 10, 50, 100 μM and was loaded at a flow rate of 20 μl/min. The binding time of saeR protein and fenoprofen was 240 s and natural dissociation time was 240 s. The analysis software used for the experimental results is TraceDrawer (Ridgeview Instruments ab, Sweden) and the analysis method is One To One analysis model.

Electrophoretic Mobility Shift Assays (EMSA). The *hla* promoter fragment was obtained by PCR from *S. aureus* ST1792. EMSA Probe Biotin Labeling Kit (Beyotime, China) was used to attach biotin labeling to the fragment. Then according to the instructions, the phosphorylated SaeR protein and biotin-labeled DNA probe were incubated in a 10 μl system at 25°C for 20min. For the fenoprofen treatment group, fenoprofen was co-incubated with SaeR protein for 20 min to enable fenoprofen to fully bind to the protein, and then DNA probes were added for further incubation for 20 min. After incubation, the mixtures were electrophoresed in 5% native polyacrylamide gel in 1X TBE buffer. Then transferred to a charged nylon membrane (Beyotime, China) in 0.5X TBE buffer. The biotin-labelled probe was detected using the Chemiluminescent EMSA Kit (Beyotime, China).

molecular dynamics (MD) simulation. Molecular dynamics simulation was based on Gromacs 2019.6 package. The water model was TIP4P, and the force field was Charmm 36. We Created dodecahedral periodic boxes with boundary conditions on three spatial dimensions and the minimum distance between the protein and the box frame was 1.2 nm. Firstly, protein energy was minimized in vacuum. Then, TIP4P model water molecules were used to fill the simulation box, and

sodium and chloride ions were added to simulate the physiological environment. The ion concentration was 150 mM, and the number of ions was adjusted to balance the system charge. The protein reenergy was minimized after the addition of fenoprofen solvent, where the skeleton atoms of the protein are confined to certain positions and the solvent diffuses freely. The coupling temperature was 310 K (about 37 °C), dispersion was corrected by EnerPres, and Parrinello-Rahman acted as the pressure controller. The reference pressure is 1.0 bar. LINCS method was used to constrain all keys, and the long range electrostatic action method was PME (Particle Mesh Ewald), and the cut-off value of electrostatic action was 1.0 nm. 100 ns simulation was performed after pre-equilibrium simulation. At the end of the simulation, 7 G data were collected. Gromacs 2019.6 was used to calculate the root mean square deviation (RMSD), mean structure, cyclotron radius and hydrogen bond of the protein.

Cytotoxicity. Osteoblastic MC3T3-E1 (GNM15, Shanghai, Cell bank of typical culture preservation Committee of Chinese Academy of Sciences, China) cells were used to detect the cytotoxicity of fenoprofen. MC3T3 cells were cultured in α-MEM (Gibco, Invitrogen, USA) supplemented with 10% fetal bovine serum (FBS, Gibco, USA). 10000 cells were added to each well of the 96-well plate and different concentrations of fenoprofen (10, 50, 100 μM) were added to the cell culture medium and incubated at 37°C in a humidified atmosphere of 5% CO2. The control group was added with the same concentration of DMSO (0.1%). After incubation for 24 h, the original cell culture medium was removed and 100 μl fresh medium containing 10% Cell Counting Kit-8 (CCK-8, Beyotime Bio-Tech, China) was added and incubated for 2 h. Then the medium was transferred to a new 96-well plate and the absorbance at 450 nm was measured using a microplate reader (BIO-TEK, ELX 800). In addition, after cells and drugs were co-incubated for 24 h, the medium was removed and the cells were washed twice with PBS (PH 7.4). Calcein/PI Cell Viability/Cytotoxicity Assay Kit (Beyotime Bio-Tech, China) was used to stain live and dead cells. Fluorescent images of cells were taken by a fluorescence microscope (Leica TCS DMI8, Germany).

***Staphylococcus aureus* growth curve assay.** The *S. aureus* were cultured overnight at 37°C and was diluted to $1x10^6$ colony units (CFU)/ml in TSB. Add 200 μl bacterial suspension containing different concentrations of fenoprofen into the 96-well plate (10, 50, 100 μM). The control group was added with the same concentration of DMSO (0.1%). The absorbance of 600 nm was measured at 0, 2, 4, 6, 8, 12, 24 h.

Bacterial cytotoxicity assay. The *S. aureus* ST1792 were treated with fenoprofen (100 μM) in TSB and incubated overnight at 37°C. The control group was added with the same concentration of DMSO (0.1%). 10000 MC3T3 cells were added to each well of the 96-well plate and incubated overnight. The next day, the bacterial suspension was centrifuged at 6000 RPM for 5min and resuspended with PBS (PH 7.4). Then each groups were adjusted to $OD_{600} = 0.8$ with PBS. Bacterial suspension was added to the cell culture medium (MOI=20:1) and incubated in a cell culture incubator (37 °C, 5% CO2) for 6 h. CCK8 was used to measure cell activity and Calcein/PI Cell Viability/Cytotoxicity Assay Kit was used to stain live and dead cells.

Hemolytic test. 1ml of fresh blood was collected from the hearts of healthy 8-week male C57 BL/6 mice and then added to the anticoagulant tube. The fresh blood was centrifuged at 4°C for 15 min

at 2500 rpm. The supernatant serum was removed and the remaining concentrated red blood cells were re-suspended with 5 ml sterile PBS (PH 7.4). Centrifugation and resuspension were repeated 5 times. Add 200 μl red cell suspension and 100 μl Bacterial suspension (OD600=0.8) to 1.5ml eppendorf tube. PBS and 0.5% TrixonX-100 were used as negative and positive controls, respectively. 700 μl PBS were add into the tubes of each group to reach 1ml and the tubes were incubated at 37° C for 4 h. After incubation, the tubes were centrifuged for 10 min, 10000 rpm at room temperature. Then the supernatant was taken and the absorbance was detected by a microplate reader (BIO-TEK, ELX 800) at a wavelength of 541 nm. Hemolysis percentage = (OD Sample - OD PBS)/(OD TrixonX-100 - OD PBS).

Fluorescent staining of intracellular bacteria. The GFP labeled wild type and *saeRS* mutant strains were maintained on Tryptic Soy Agar (TSA) plates with 10 μg/ml chloramphenicol. The GFP labeled ST1792 was treated with different concentrations of fenoprofen (10, 50, 100 μM) in TSB containing 10 μg/ml chloramphenicol at 37 °C for 24 h. The other groups were added with the same concentration of DMSO (0.1%). MC3T3 cells were seeded in the 24-well plates at a density of 1×10^5 cells/well. After 1 day incubation, cells were infected with GFP labeled *S. aureus* at a concentration of 1×10^5 CFU/ml. 1 h later, MC3T3 cells were treated with 50 μg /ml of gentamicin for 1 h to kill extracellular bacteria. The cells were washed three times with sterile PBS (PH 7.4) and then fixed with 4% paraformaldehyde for 15min. After being permeabilized by PBS with 0.1% Triton X-100 solution for 10 min, the cells were stained with TRITC Phalloidin (Yeasen, Shanghai, China) and DAPI (Invitrogen, Carlsbad, CA, USA). Images were acquired with a fluorescence microscopy (Leica TCS DMI8, Germany). To quantify the ratio of *S. aureus* internalization in MC3TC osteoblastic cells, three different microscopic fields under 4×10 magnification were randomly acquired for each sample, the ratio of the number of cells with *S. aureus* to the total cell number was quantified by Image J.

Internalized *S. aureus* CFU counting. Osteoblastic MC3T3 cells were cultured in α-MEM supplemented with 10% FBS. MC3T3 cells were seeded in the 24-well plates at a density of 1×10^5 cells/well. After 1 day incubation, cells were infected with GFP labeled strains treated or untreated with fenoprofen at a concentration of 1×10^5 CFU/ml. 1 h later, MC3T3 cells were treated with 50 μg /ml of gentamicin for 1 h to kill extracellular bacteria. The cells were washed three times with sterile PBS and were lysed with 0.1% Triton X-100 solution to release all intracellular bacteria. A series of diluents of the cell lysates were spread on a sheep blood agar plate and incubated overnight at 37°C. The relative number of bacterial CFU was calculated by dividing the number of CFU by the total number of MC3T3 cells.

Biofilm formation and biomass. *S. aureus* strains were cultured overnight at 37°C and was diluted to $1x10^6$ colony units (CFU)/ml by TSBg. Add 100 μl bacterial suspension treated or untreated with fenoprofen into the 96-well plate and incubated overnight at 37°C. After 1 day incubation, the biofilms in the plate were gently washed by sterile PBS (PH 7.4) three times, 100 μl methanol was added to each well to immobilize the biofilms for 15 min, then 100 μl 0.1% crystal violet ethanol solution was added and stained for 15 min. The biofilms after crystal violet staining were then imaged by camera. Then 100 μl 33% acetic acid was added to the well to dissolve crystal violet and the corresponding absorbance of crystal violet solution at 590 nm was measured to evaluate the

ability of fenoprofen on biofilm formation inhibition.

Overnight cultured *S. aureus* strains were serially diluted to $1x10^6$ colony units (CFU)/ml by TSBg. 1ml of bacteria suspension containing different concentrations of fenoprofen were incubated overnight in a 24-well tissue culture plate at 37 °C. According to the illustration of the Live/Dead BacLight bacteria viability kits (L13152, Invitrogen, USA), the biofilm after fenoprofen treatment was gently rinsed three times with PBS, and then stained with 500 µl staining reagent mixtures for 30 min in darkness. Lastly, the biofilm was observed by CLSM (Leica TCS SP8, Germany). The green fluorescent represented the live bacteria.

Primary attachment assay. *S. aureus* strains were cultured overnight at 37°C and was diluted to $1x10^6$ colony units (CFU)/ml by TSBg. Add 1 ml bacterial suspension treated or untreated with fenoprofen into the 12-well polystyrene flat-bottom plate or 12-well wells containing titanium plates and incubated at 37°C for 2 h. Subsequently, the spent media supernatant in each well was aspirated, and the wells were washed three times with sterile PBS (PH 7.4) to remove the unattached *S. aureus*. The attached bacteria on polystyrene or titanium surfaces were washed with TSB and subjected to CFU counting. The attachment proportion was calculated by dividing the number of attached bacteria by the initial bacterial count.

eDNA, Protein, and PIA Content in Biofilms assay. *S. aureus* strains were cultured overnight at 37°C and was diluted to $1x10^6$ colony units (CFU)/ml by TSBg. Add 1 ml bacterial suspension treated or untreated with fenoprofen into the 6-well plate and incubated overnight at 37°C. After 1 day incubation, the biofilms in the plate were gently washed by sterile PBS (PH 7.4) three times, and the washed biofilms were collected in 1 mL of PBS. The collected biofilms were then filtered through a 0.22 µm filter. To assess the content of eDNA in biofilms, Nanodrop 2000c Spectrophotometer (Thermo Fisher Scientific, USA) was used to measure the content of eDNA. SYTOX (Invitrogen S34860, USA) was used for staining eDNA in the biofilms, and CLSM (Leica TCS SP8, Germany) was used to observe eDNA in the biofilms. To assess the content of protein in biofilms, BCA Protein Assay Kit (Biyuntian, Shanghai, China) was used to measure the content of protein in the biofilms. To assess the content of PIA in biofilms, the dot blot assay was performed to measure the the content of PIA in the biofilms with the WGA-horseradish peroxidase (HRP) conjugate (Sigma-Aldrich, L3892, USA).

Neutrophil and macrophage experiments. Neutrophils were isolated from the blood of healthy volunteers and the protocol was approved by the Ethics Committee of Shanghai Jiaotong University Affiliated Sixth People's Hospital. Each volunteer signed a written informed consent. Neutrophils were isolated from blood using the Human Peripheral Blood Neutrophil separation Kit (Solarbio, Beijing, China) and according to the instructions. Macrophage RAW264.7 cells were from Shanghai Cell Bank of typical Culture Preservation Committee of Chinese Academy of Sciences. Neutrophils were cultured in RPMI-1640 medium containing 10% fetal bovine serum (FBS) and Macrophages were cultured in DMEM medium containing 10% FBS.

For leukocytes infiltration of the biofilm, *S. aureus* strains first formed biofilms in TSBg in 24-well plates for 1 day. WT+Fen group was added with 100 µM fenoprofen and other groups were added with the same concentration of DMSO (0.1%). After 1 day of incubation, the biofilms were stained with the Live/Dead BacLight Bacteria Viability kit. M1 Macrophage RAW264.7 cells were

stained with CellTrace™ Violet Cell Proliferation Kit (C34571, Invitrogen™, Thermo Fisher Scientific). 1 x 10^5 stained macrophages were added into each well of the 24-well plate and co-incubated with stained biofilms for 2 h at 37°C. Then the biofilms were washed twice with sterile PBS (PH 7.4) to remove macrophages that did not infiltrate into the biofilms. The washed biofilms were imaged by CLSM (Leica TCS SP8, Germany) using three confocal channels (405 nm laser for the macrophages, 488 nm and 552 nm for the live/dead stained biofilms).

For analysis of the killing efficiency of M1 macrophages and neutrophils against biofilms and planktonic *S. aureus*, biofilms were grown in TSBg for 24 h in the 24-well plate treated or untreated with fenoprofen (100 μM) and planktonic *S. aureus* strains were grown in TSB overnight in the 24-well plate treated or untreated with fenoprofen (100 μM). For the killing efficiency of macrophages and neutrophils against biofilms, the biofilms were washed twice with sterile PBS (PH 7.4) and added 1 x 10^5 macrophages or neutrophils. The bacterial survival rate was measured by CFU counting results at 0, 30, 60, 90 min. For the killing efficiency of macrophages and neutrophils against planktonic bacteria, overnight cultured bacteria suspension were adjusted to OD_{600}=0.8 with TSB. 10 μl bacterial suspension was mixed with 990 μl cell culture medium containing 1 x 10^6 macrophages or neutrophils and added to the 24-well plate, incubating at 37°C. The bacterial survival rate was measured by CFU counting results at 0, 30, 60, 90 min. Three technical replicates were undertaken for each group.

Periprosthetic Joint Infection (PJI) model. The *S. aureus* strains were cultured overnight at 37°C and adjusted to OD_{600}=0.8 with TSB. Healthy 6-week-old male C57 BL/6 mice were randomly divided into four groups (WT, *ΔsaeRS*, WT+Fen, WT+Ibu). Mice were anesthetized with 1% pentobarbital sodium (provided by The Animal Center of Shanghai Sixth People's Hospital) and then their knees were disinfected with 75% alcohol. We dissected the knee and exposed the tibia of the mice. 0.3mm*0.8mm insulin needles (Omnican, BRAUN, Germany) were inserted into the tibia of mice as implant. We sutured the skin and injected bacterial suspension (1 x 10^6 c.f.u.) into the knee joint of the mice. Mice in WT+Fen group received intraperitoneal injections with a total dose of 100mg/kg fenoprofen everyday, mice in WT+Ibu group received intraperitoneal injections with a total dose of 100mg/kg Ibuprofen everyday and mice in other groups were intraperitoneally injected with PBS. At 1,2,3,5,7 days after infection, joint rinse solution, joint soft tissue, joint bone tissue and implant were harvested and count the bacterial burden by CFU counting. At 4 weeks after infection, the tibia of the mice was collected for X-ray (XRD; Rigaku Ultima IV) and micro-CT scanning (Skyscan 1172, Bruker Micro-CT, Germany) at a resolution of 9 μm. Cancellous bone mineral density (BMD), bone volume/total volume (BV/TV) and trabecular thickness (TB.TH) of each group were analyzed by the CTAn program (Skyscan Company, Bruker Micro-CT, Germany). Furthermore, the tibia of the mice were harvested, fixed with 4% paraformaldehyde, embedded in paraffin, cut into sections, and stained with Giemsa staining to assess infection.

Gait Analysis Experiment. Gait analysis experiment was performed on PJI model mice one week after infection. The mice were placed on the runway of the Gait analysis device and ran from start to finish. High-speed camera at the bottom of the device was used to shoot rat footprints in 120 fps, 1/4 CCD. Support time, stride length, average intensity (average pressure on the runway) and average speed were analyzed by gait analysis software. According to the pressure of each foot contacting with the ground, 3D reconstruction of footprints was performed using gait analysis

software.

Implant-associated Biofilm Infection Model. As previous describe, healthy 6-week-old male C57 BL/6 mice were divided into four groups (WT, *ΔsaeRS*, WT+Fen, WT+Ibu) and anesthetized with 1% pentobarbital sodium. Skin on posterior upper backs was shaved and disinfected with 75% alcohol. An incision approximately 1 cm was made in the back of the mice using sterile surgical scissors and titanium plates (1 ×1 cm^2 square) were inserted into the subcutaneous. The wound was carefully sutured and prepared suspension (1 x 10^7 c.f.u.) was injected on the surface of implant. Luminescent strain ST1792-LUX was used for real-time monitoring implant-associated infection via bioluminescence imaging at 1, 2, 3, 5, 7 days after infection. Mice in WT+Fen group received intraperitoneal injections with a total dose of 100mg/kg fenoprofen everyday, mice in WT+Ibu group received intraperitoneal injections with a total dose of 100mg/kg Ibuprofen everyday and mice in other groups were intraperitoneally injected with PBS. At 1, 3 and 7 days after infection, mice were killed, implant and surrounding infected soft tissues were harvested. For CLSM images of biofilms on the implant surface, the implant was washed twice with sterile PBS (PH 7.4) immediately after it was collected from the mice vivo and the Live/Dead BacLight bacteria viability kit was used to stain the biofilm. After staining, images of the biofilms formed on the surface of the implant were taken by CLSM. For pathological sections, implant surrounding infected soft tissues of each group were harvested at 1, 3 and 7 days after infection, fixed with 4% paraformaldehyde, embedded in paraffin, cut into sections, and stained with H&E and Giemsa staining. For bacterial CFU counting, at 1,3 and 7 days after infection, the implant and surrounding infected soft tissues were subjected to high-speed grinding to transfer the bacteria to sterile PBS (PH 7.4). Then SPM methods were used to detect bacterial burden of implant and surrounding infected soft tissues. For SEM images of biofilms, at 1,3 and 7 days after infection, the implants of each group were collected and then washed gently with sterile PBS (PH 7.4) twice. The implants were fixed with 2.5% glutaraldehyde at 4°C overnight and dehydrated using a graded ethanol series (50, 70, 80, 90, 95, 99 and 100% v/v) for 10 min, freeze-dried, coated with platinum. SEM (Magellan 400, FEI, United States) was used to observe the biofilms on the surface of implants.

Construction and identification of Lys2-eGFPF mice. Briefly, upstream and downstream homologous regions of Lys2 gene were amplified by PCR from mouse DNA and "2A-EGFPF" fragment was inserted to construct Embryonic Stem cell (ES cell) target plasmids. After plasmid construction, PCR and sequencing were performed to identify whether the constructed plasmid was correct. After PCR identification, the target plasmid was extracted and transferred into the ES cells of C57BL/6 mice by electricity. Then, ES cells were screened and identified by PCR sequencing and Southern blot analysis. The identified ES clones were injected into mouse blastocysts by microinjection technique, and the blastocysts were implanted into surrogate mice to obtain F0 generation mice. KI-positive mice were crossbred with wild-type mice to obtain heterozygous F1 mice with Neo gene deleted. The homozygous F2 generation was obtained by self-crossing and screening of hybrid mice. The mouse tail tissue was cut and digested by the kit to extract genomic DNA. Primers were designed according to the inserted eGFPF and upstream and downstream sequences, and the PCR amplification products were identified by electrophoresis. Meanwhile, the blood of mice was collected and the fluorescence of immune cells in the blood was detected by flow cytometry.

CLSM images of biofilms and leukocytes in Lys2-eGFPF mice. As described before, Lys2-eGFPF mice and *S. aureus* strains with mcherry plasmid were used to construct implant-associated biofilm infection model. At 3 days after infection, the implants on the back of the mice were collected and washed twice with sterile PBS (PH 7.4). The washed implants were imaged by CLSM (Leica TCS SP8, Germany) immediately. Myeloid derived immune cells of Lys2-eGFPF mice showed green fluorescence and *S. aureus* showed red fluorescence.

Resistance Induction Assay. *S. aureus* ST1792 was treated with fenoprofen (100 μM) for 24h, and the control group was added with the same concentration of DMSO (0.1%). 24h later, 10 μl of fenoprofen-treated *S. aureus* was added into two groups of 5 ml TSB, fenoprofen (final concentration was100 μM) was added into one group, and DMSO (final concentration was 0.1%) was added into the other group with the same concentration, which were marked as fenoprofen-treated group and control group at day 2. This method was followed up to 42 days, and the RNA of *S. aureus* was extracted on each day to detect the expression of SaeP and hla. Fold change was the ratio of gene expression between the fenoprofen-treated group and the control group. Three repetitions were performed at each time point. DNA was extracted from the bacteria on day 7, day 14, day 21, day 28, day 35, and day 42, respectively, and PCR and sequencing of saeR were performed to verify if spontaneous mutation occurred in the saeR sequence.

Supplementary Figures:

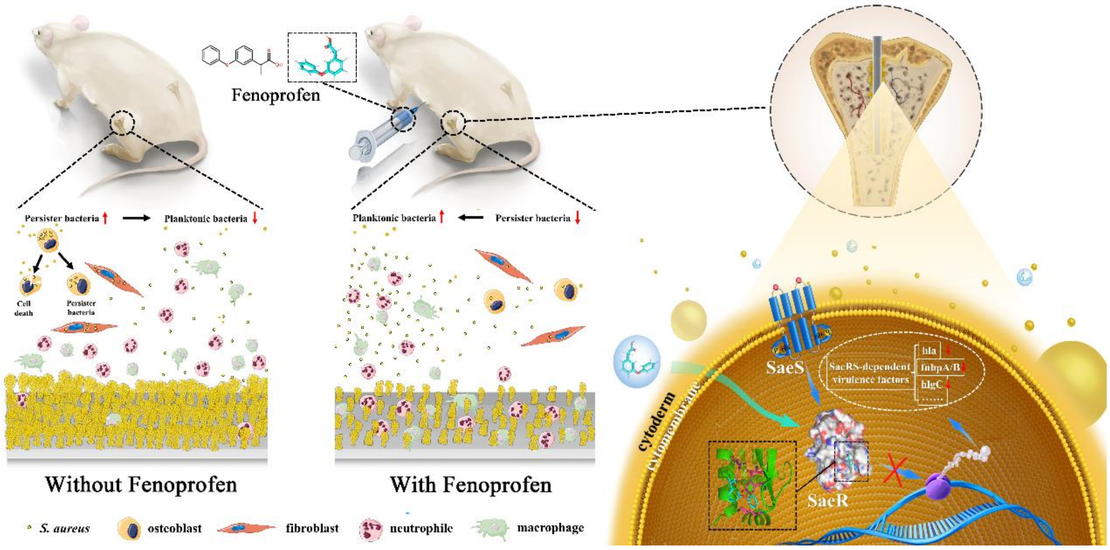

Supplementary Figure 1. Schematic diagram of fenoprofen for the treatment of orthopedic implant-associated infection. Implant-associated infected mice are intraperitoneally injected with fenoprofen. When fenoprofen enters bacteria, it can bind to the promoter binding region of SaeR protein, inhibit transcriptional activation of downstream virulence factors such as *hla*, *fnbpA/B* and *hlgC*, attenuate the internalization of *S. aureus* and cause the *S. aureus* biofilms on implant surfaces to become sparse and porous, ultimately enabling host immune system to clear planktonic bacteria and eliminate biofilm more effectively.

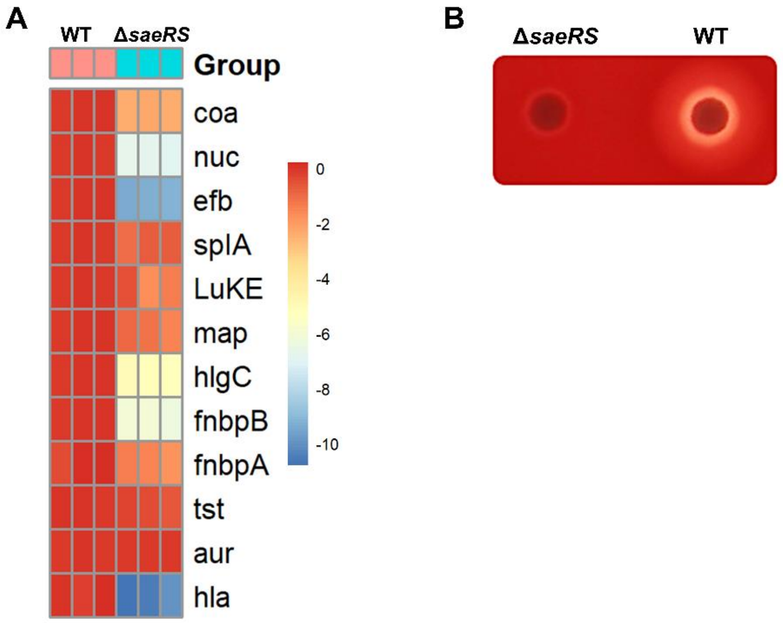

Supplementary Figure 2. The virulence expression and hemolytic ability of *saeRS* mutant strains were decreased. (A) qPCR results of the virulence expression of the WT and *saeRS* mutant strains. (**B**) Hemolytic phenotype comparative analyses of the WT and *saeRS* mutant strains in sheep blood agar plates.

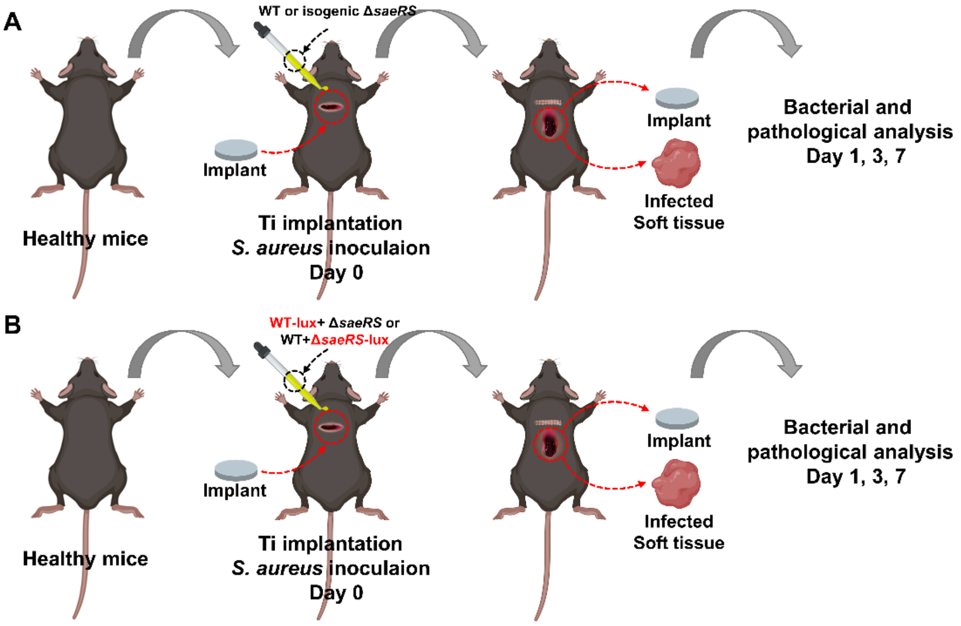

Supplementary Figure 3. Schematic of mouse implant-associated biofilm infection model construction. (A) Schematic of mouse implant-associated biofilm infection model construction. The surface of the mouse implant was inoculated with 1 x 10^7 CFU luminescent wild-type or isogenic *saeRS* mutant strains. (**B**) Schematic of mixed implant-associated infection model construction. Mice were subcutaneously injected with 1 x 10^7 CFU *S. aureus* (WT-lux/Δ*saeRS* or WT/Δ*saeRS*-lux, WT/*saeRS* mutant = 1:1).

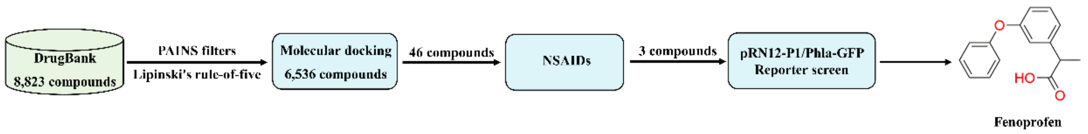

Supplementary Figure 4. Procedure for virtual screening. Flow chart of the virtual screening procedure based on the structure of the SaeR protein in drugbank database.

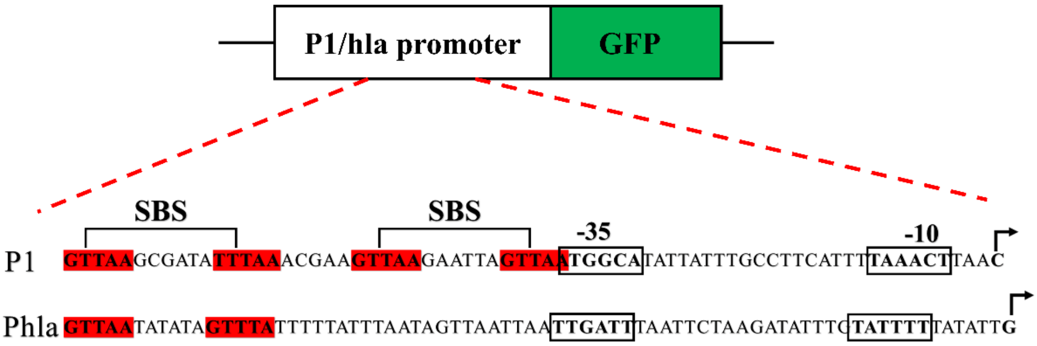

Supplementary Figure 5. Schematic diagram of P1/Phla-GFP reporter plasmid construction. Schematics of the pRN12-P1/Phla-GFP reporter system construction. The red sequence represents SaeR-binding sequences (SBSs). Arrows indicate the start sites of transcription. The promoter sequence are shown in boxes. Phla, the promoter of α-hemolysin (*hla*).

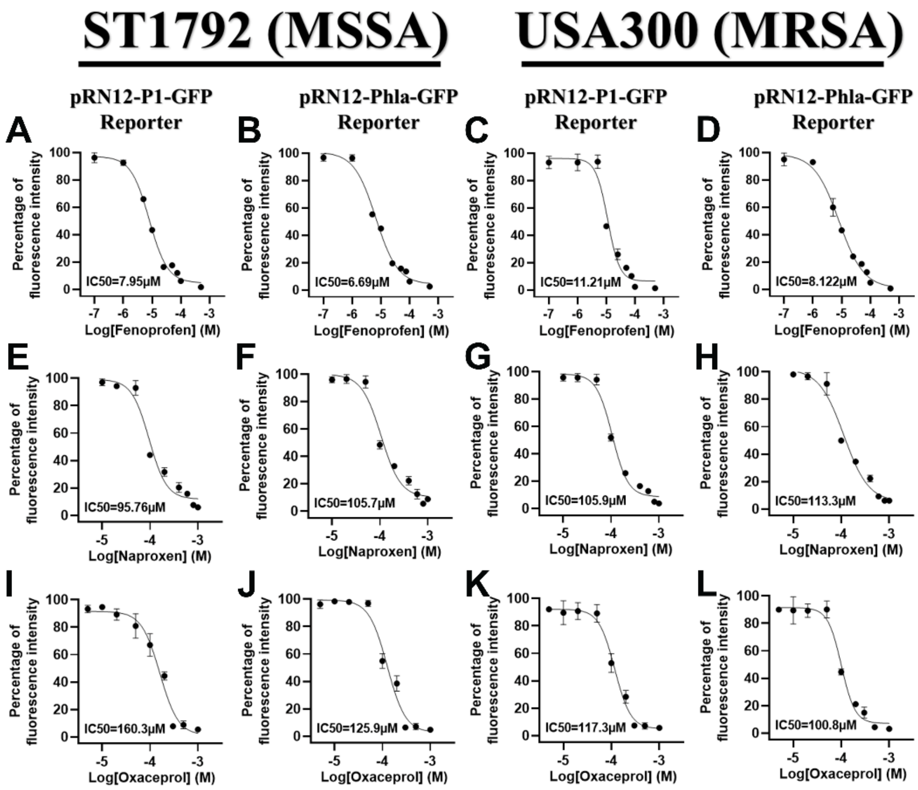

Supplementary Figure 6. Efficiency of fenoprofen in inhibiting transcriptional activation of SaeR protein. (**A, E, I**) The suppression efficiency of Fenoprofen, Naproxen, and Oxaceprol was detected using the pRN12-P1-GFP reporter system in ST1792 (MSSA). (**B, F, J**) The suppression efficiency of Fenoprofen, Naproxen, and Oxaceprol was detected using the pRN12-Phla-GFP reporter system in ST1792 (MSSA). (**C, G, K**) The suppression efficiency of Fenoprofen, Naproxen, and Oxaceprol was detected using the pRN12-P1-GFP reporter system in USA300 (MRSA). (**D, H, I**) The suppression efficiency of Fenoprofen, Naproxen, and Oxaceprol was detected using the pRN12-Phla-GFP reporter system in USA300 (MRSA).

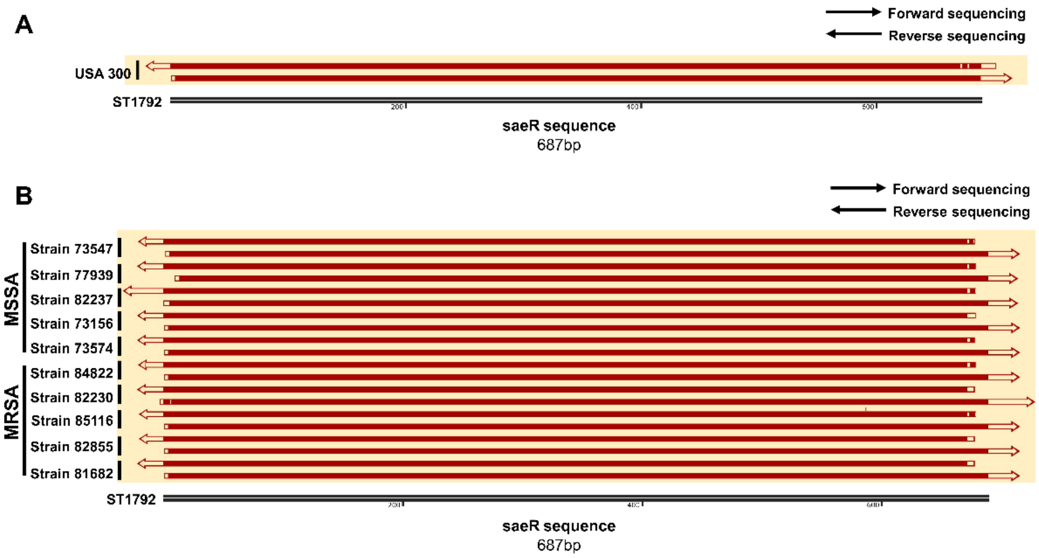

Supplementary Figure 7. The saeR sequence of Staphylococcus aureus is highly conserved. (**A**) The DNA was extracted from USA300 and ST1792. Then the DNA was performed PCR and the saeR sequence was sequenced. (**B**) The DNA was extracted from clinical *S. aureus* strain (5 MSSA and 5 MRSA). Then the DNA was performed PCR and the saeR sequence was sequenced.

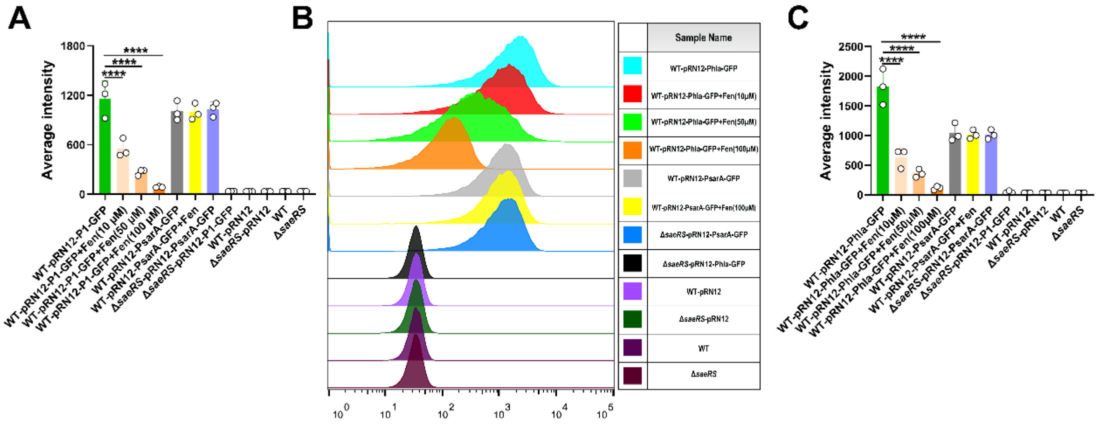

Supplementary Figure 8. Fenoprofen attenuates the ability of saeR protein to activate promoters. (**A**) Flow data quantitative analysis of the efficiency of fenoprofen on pRN12-P1-GFP reporting system. Each column shows the mean ± SD of three independent experiments and analyzed by the one-way ANOVA. ****P<0.001. (**B**) Flow data of the effect of fenoprofen on pRN12-Phla-GFP reporting system. (**C**) Flow data quantitative analysis of the efficiency of fenoprofen on pRN12-Phla-GFP reporting system. Each column shows the mean ± SD of three independent experiments and analyzed by the one-way ANOVA. ****P<0.001.

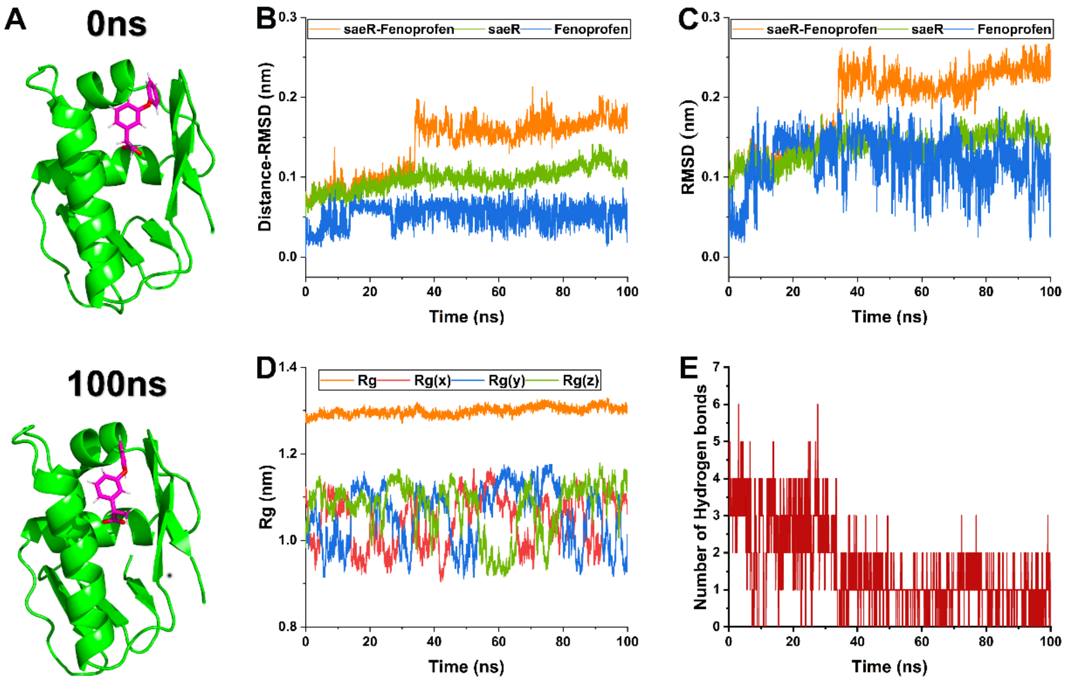

Supplementary Figure 9. Molecular dynamics (MD) simulation results of SaeR-fenoprofen complex. (**A**) Conformational changes of SaeR-fenoprofen in 100 ns molecular dynamics simulations, showing that fenoprofen has been stably bound to the SaeR functional domain. (**B**) Root mean square deviation (RMSD) values of heavy atoms with distance in all models. (**C**) Root mean square deviation (RMSD) values of heavy atomic skeleton in all models. (**D**) The gyration radius of SaeR-fenoprofen complex. (**E**) Dynamic statistics of hydrogen bonding between SaeR protein and fenoprofen in 100 ns molecular dynamics simulations.

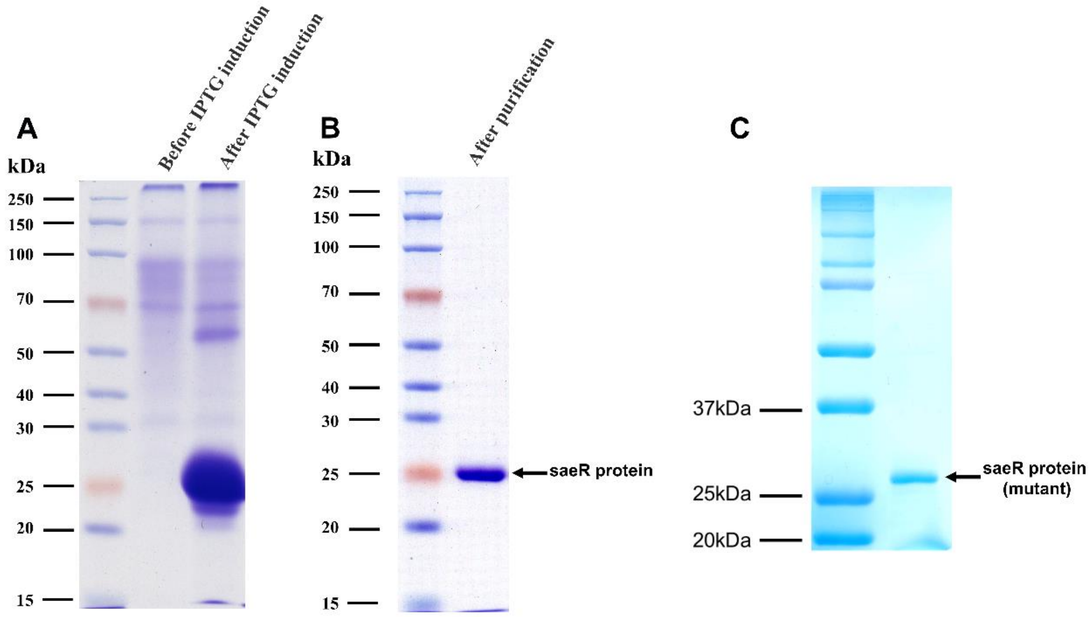

Supplementary Figure 10. Expression and purification of SaeR protein. (**A**) SDS-PAGE protein gel image of 6His-saeR protein before and after IPTG induction. (**B**) SDS-PAGE protein gel image of 6His-saeR protein after purification. (**C**) SDS-PAGE protein gel image of 6His-saeR mutant

protein after purification.

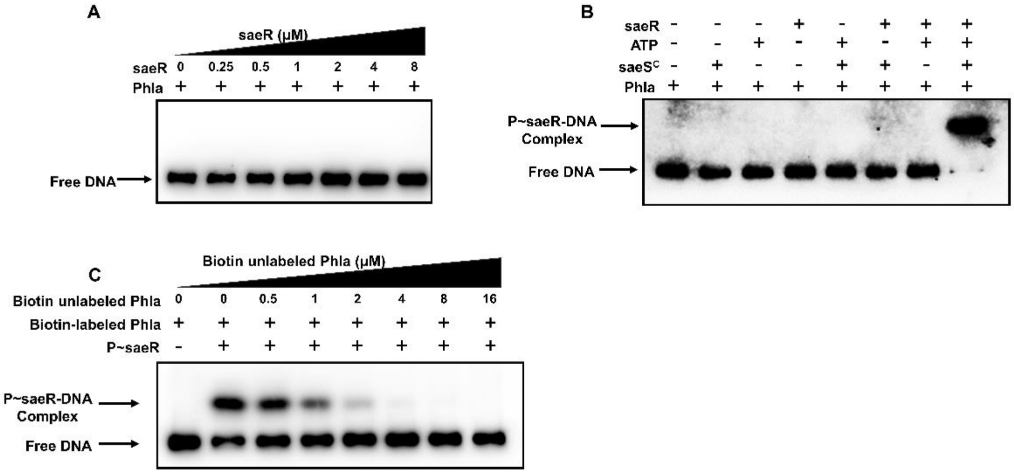

Supplementary Figure 11. The saeR protein needs to be phosphorylated to bind to the *hla* promoter and this binding is specific. (**A**) EMSA of unphosphorylated SaeR with a biotin-labeled *hla* promoter fragment. The arrow indicates free DNA. (**B**) EMSA of SaeS^{C}, unphosphorylated SaeR, ATP, and their mixtures with a biotin-labeled *hla* promoter fragment. The arrow indicates free DNA and the protein–DNA complex. (**C**) EMSA of phosphorylated SaeR with biotin-labeled *hla* promoter fragments and biotin-unlabeled *hla* promoter fragments. The arrow indicates free DNA and the protein–DNA complex.

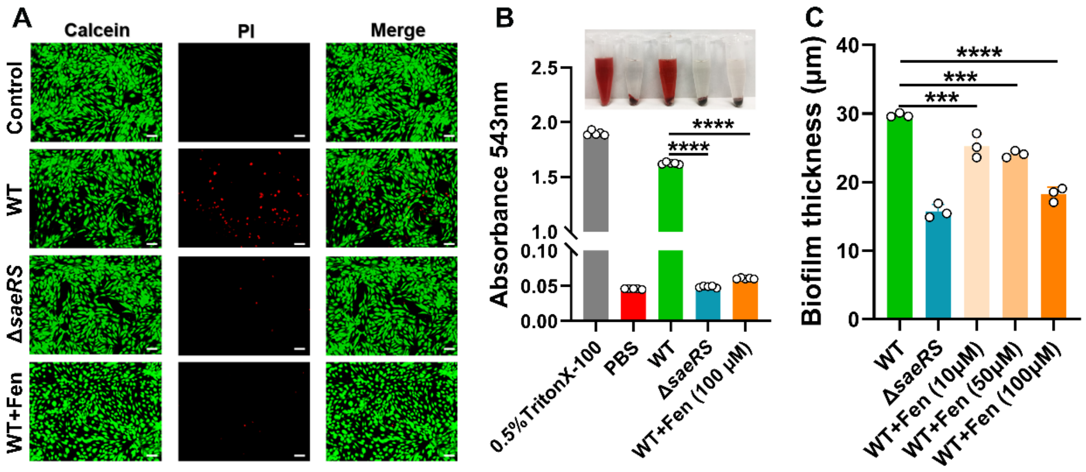

Supplementary Figure 12. Effect of fenoprofen on toxicity, hemolysis and biofilm formation of *S. aureus*. (**A**) Live/dead staining of MC3T3 cells treated with *S. aureus*. Green fluorescence represents living cells and red represents dead cells. (**B**) Representative images and data results of hemolysis tests. Each column shows the mean ± SD of three independent experiments and analyzed

by one-way ANOVA. ****P<0.0001. (**C**) Biofilm thickness of CLSM images. Each column shows the mean ± SD of three independent experiments and analyzed by the one-way ANOVA. ***P<0.001, ****P<0.0001.

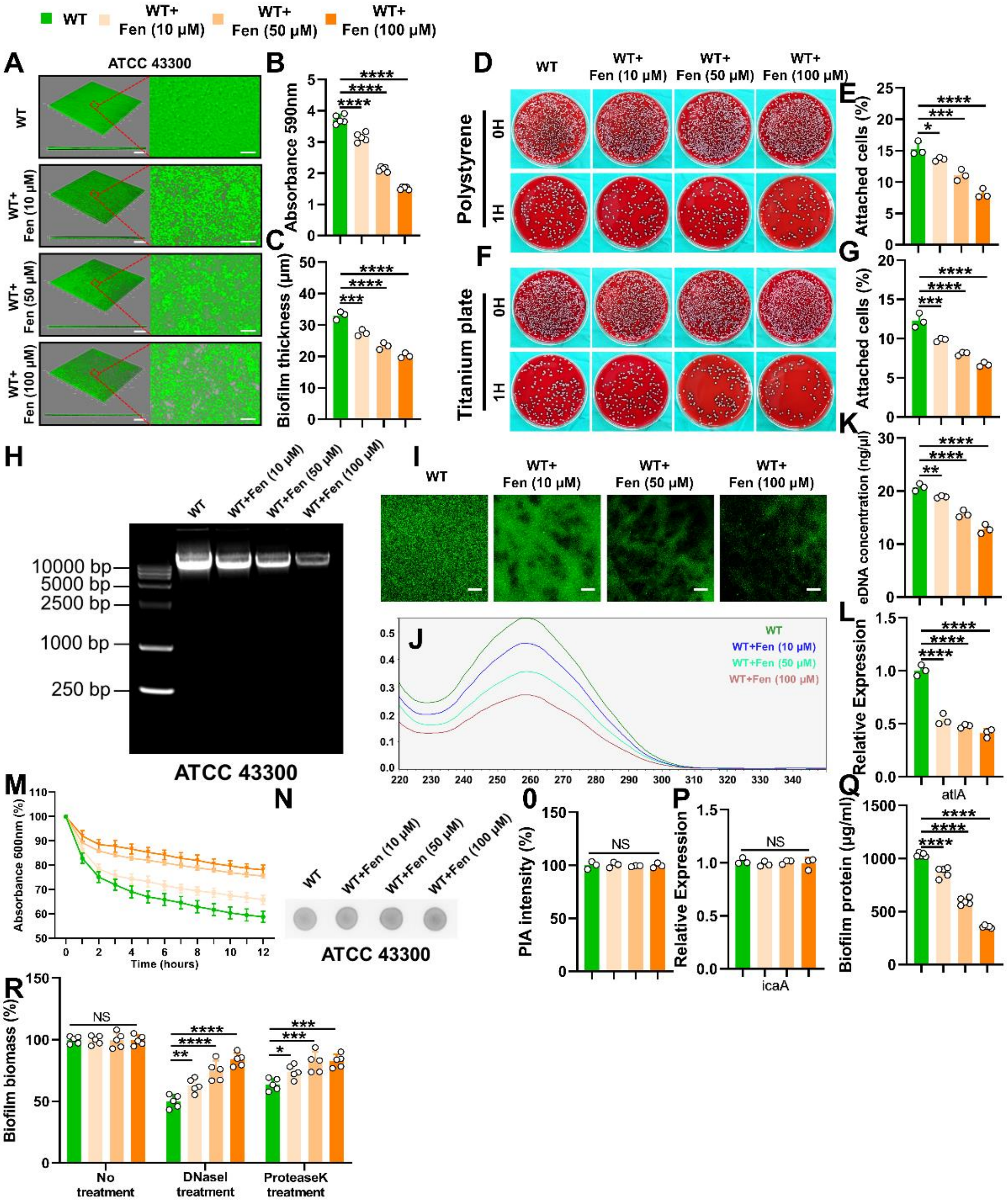

Supplementary Figure 13. The effect of fenoprofen on the adhesion ability, biofilm formation, and biofilm matrix of *S. aureus* ATCC 43300. (**A**) CLSM reconstructed images of biofilms from each group. (**B**) Crystal violet staining results of biofilms from each group (n=5). (**C**) The thickness results of the biofilm formed by Staphylococcus aureus after treatment with fenoprofen. (**D** to **G**)

Spread plate results of the inhibitory effect of fenoprofen on the adhesion ability of *S. aureus* ATCC 43300 to polystyrene (D) and Titanium plate (F) and the corresponding CFU counting results (E and G) (n=3). (**H**) Agarose gel electrophoresis images of eDNA in the biofilms of each group. (**I**) SYTOX staining images of eDNA in the biofilms of each group. (**J** and **K**) Absorbance curve images and quantitative results of eDNA in the biofilms of each group (n=3). (**L**) The effect of fenoprofen on the expression of atlA gene in *S. aureus* ATCC 43300 (n=3). (**M**) The effect of fenoprofen on the Triton-X100 induced autolysis rate (n=3). (**N** and **O**) Dot blot images and corresponding semiquantitative results of PIA in the biofilms of each group (n=3). (**P**) The effect of fenoprofen on the expression of icaA gene in *S. aureus* ATCC 43300 (n=3). (**Q**) Quantitative results of protein in the biofilms of each group (n=5). (**R**) The effects of DNase I and protease K on the biofilm formed by fenoprofen-treated and untreated *S. aureus* (n=5). Scale bars, 200 μm and 40 μm (A), 200 μm (I).All results are presented as the means ± SDs. *P<0.05, **P<0.01, ***P<0.001, ****P<0.0001, and data were analyzed by one-way ANOVA (B, C, E, G, K, L, O, P, Q, R).

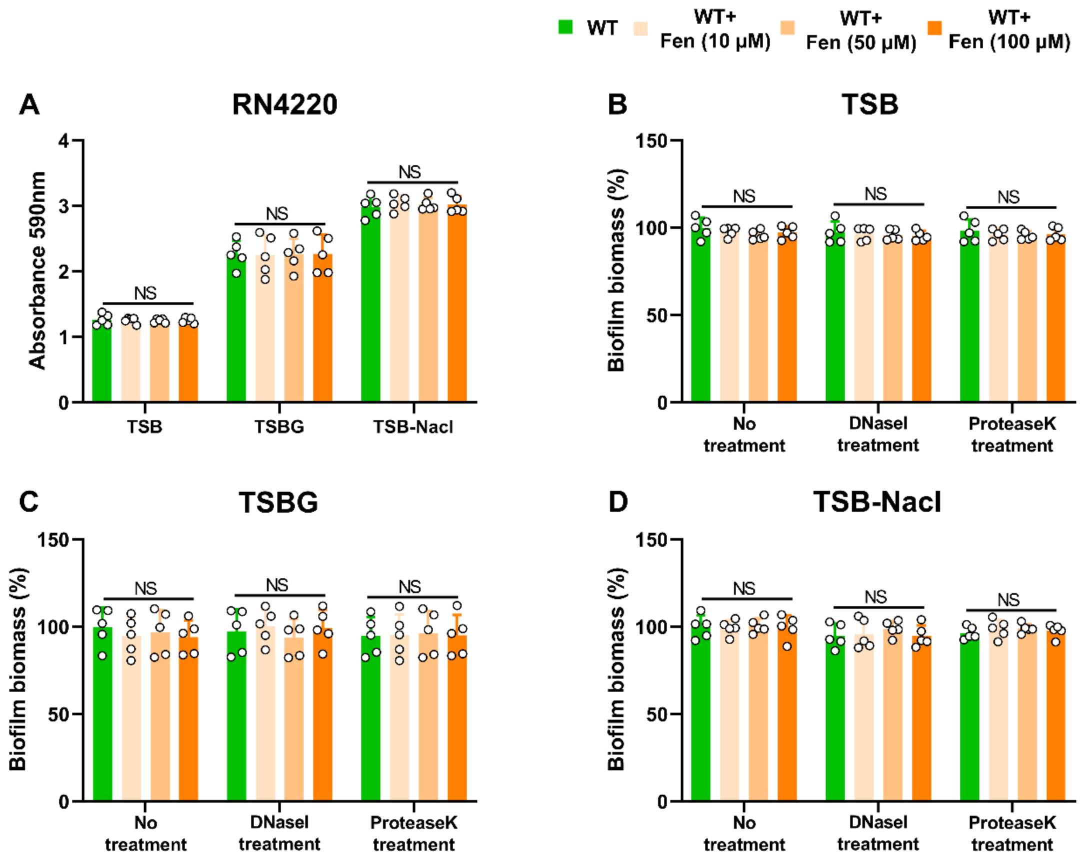

Supplementary Figure 14. Anti-biofilm activity of fenoprofen against *S. aureus* RN4220 strain.

(**A**) Crystal violet staining results of biofilms from each group in TSB, TSBG, and TSB-Nacl (n=5). (**B-D**) The effects of DNase I and protease K on the biofilm formed by fenoprofen-treated and untreated *S. aureus* RN4220 (n=5).

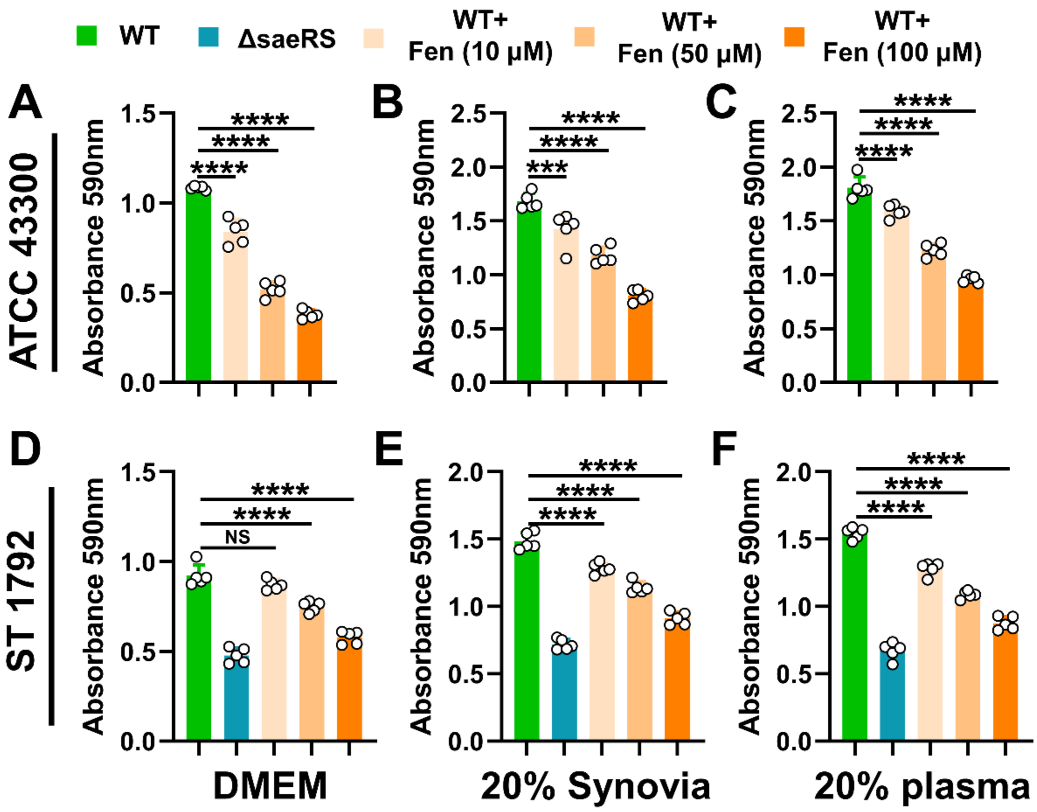

Supplementary Figure 15. The anti-biofilm effect of fenoprofen on *S. aureus* ATCC 43300 and ST1792 under different culture conditions. (**A** and **D**) The anti-biofilm effect of fenoprofen on *S. aureus* ATCC 43300 (A) and ST1792 (D) in DMEM (n=5). (**B** and **E**) The anti-biofilm effect of fenoprofen on *S. aureus* ATCC 43300 (B) and ST1792 (E) in 20% synovia (n=5). (**C** and **F**) The anti-biofilm effect of fenoprofen on *S. aureus* ATCC 43300 (C) and ST1792 (F) in 20% plasma (n=5). All results are presented as the means ± SDs. ***P<0.001, ****P<0.0001, and data were analyzed by one-way ANOVA (A to F).

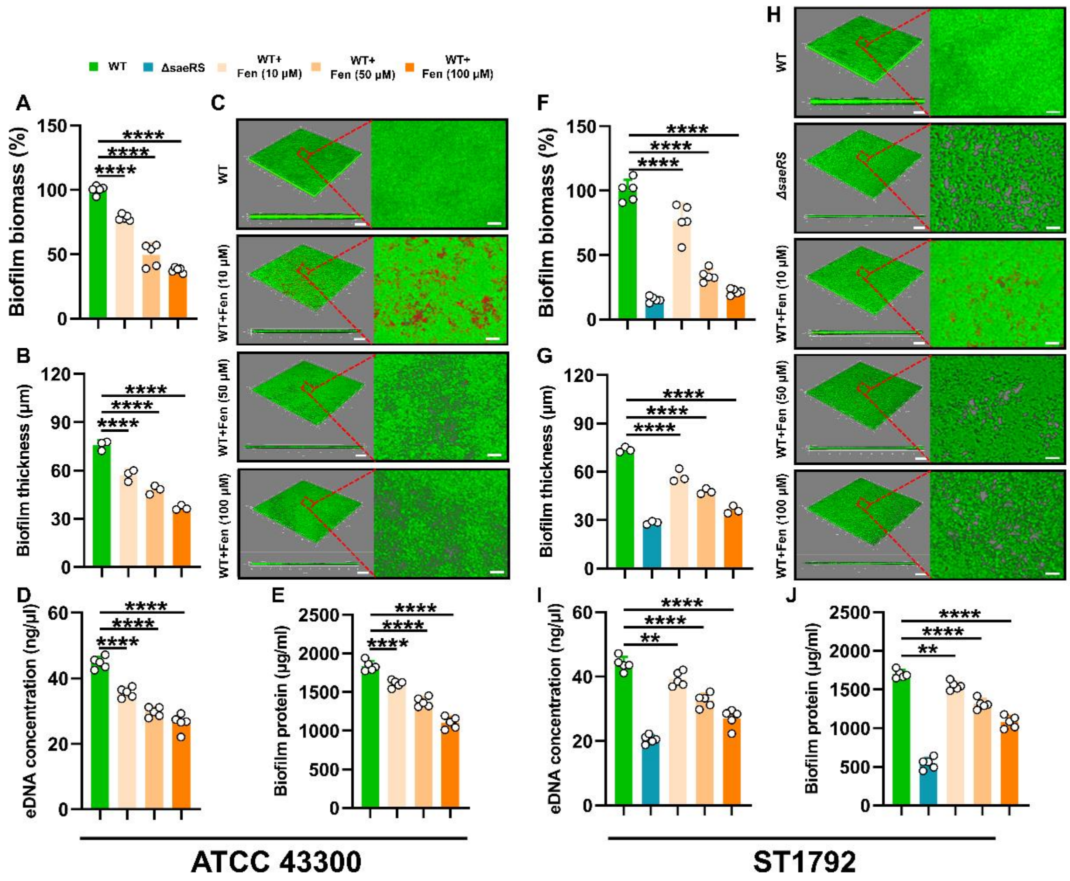

Supplementary Figure 16. Fenoprofen prevents pre-formed biofilms from maturing. (**A** to **C**) CLSM reconstruction images and crystal violet staining quantitative data of the pre-formed biofilms of *S. aureus* ATCC 43300 after treatment with fenoprofen for two days (n = 5). (**D** and **E**) The contents of eDNA and protein in the biofilms of *S. aureus* ATCC 43300, which were pre-formed and then treated with fenoprofen for two days (n = 5). (**F** to **H**) CLSM reconstruction images and crystal violet staining quantitative data of the pre-formed biofilms of *S. aureus* ST1792 after treatment with fenoprofen for two days (n = 5). (**D** and **E**) The contents of eDNA and protein in the biofilms of *S. aureus* ATCC 43300, which were pre-formed and then treated with fenoprofen for two days (n = 5). Scale bars, 200 μm and 40 μm (C and H).All results are presented as the means ± SDs. *P<0.05, **P<0.01, ***P<0.001, ****P<0.0001, and data were analyzed by one-way ANOVA (A, B, D, E, F, G, I, J).

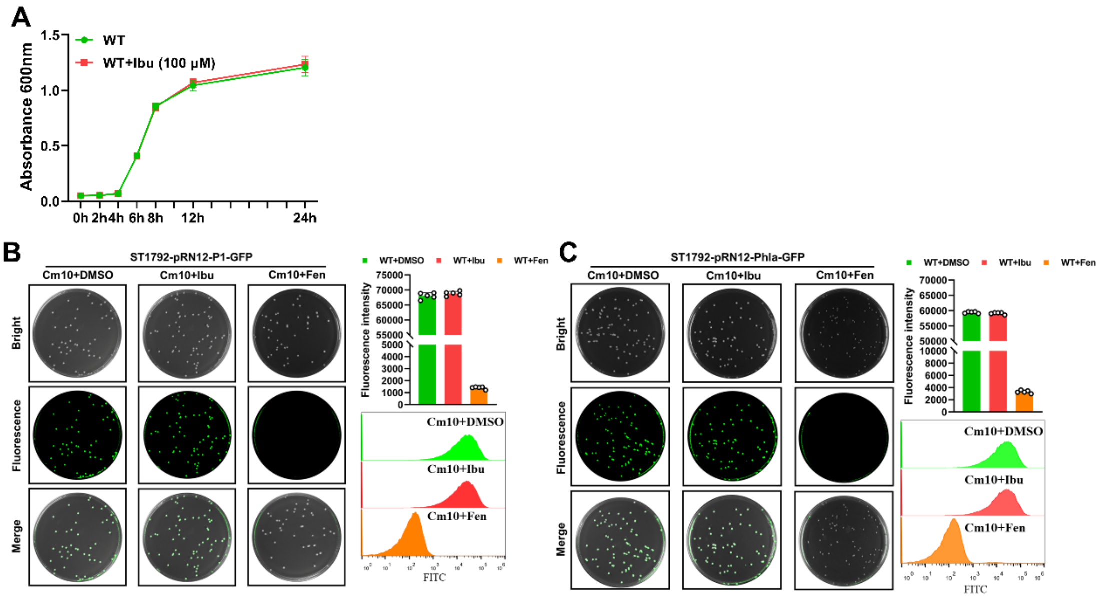

Supplementary Figure 17. Ibuprofen is not an inhibitor of *S. aureus* saeR protein. (A) Ibuprofen did not affect the growth ability of *S. aureus*. (**B** and **C**) Effects of ibuprofen on fluorescent reporter strains (ST1792-pRN12-P1-GFP and ST1792-pRN12-Phla-GFP), the results showed that ibuprofen did not inhibit the activation of *saeP* promoter and *hla* promoter (n = 5).

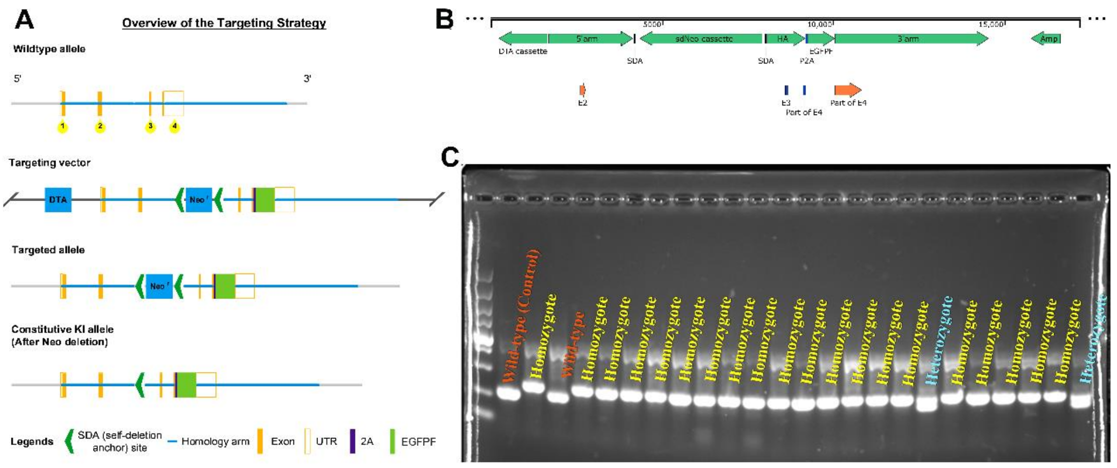

Supplementary Figure 18. Construction and identification of Lys2-eGFPF mice. (**A**) The overview of the construction strategy. For the KI model, the TGA stop codon was replaced with the “2A-EGFPF” cassette. In the targeting vector, the Neo cassette was flanked by SDA (self-deletion anchor) sites. DTA will be used for negative selection. (**B**) Schematic diagram of lyz2-eGFPF target plasmid design. (**C**) The mice were identified by PCR. The wild-type is shown in orange, the homozygote in yellow, and the heterozygote in blue.

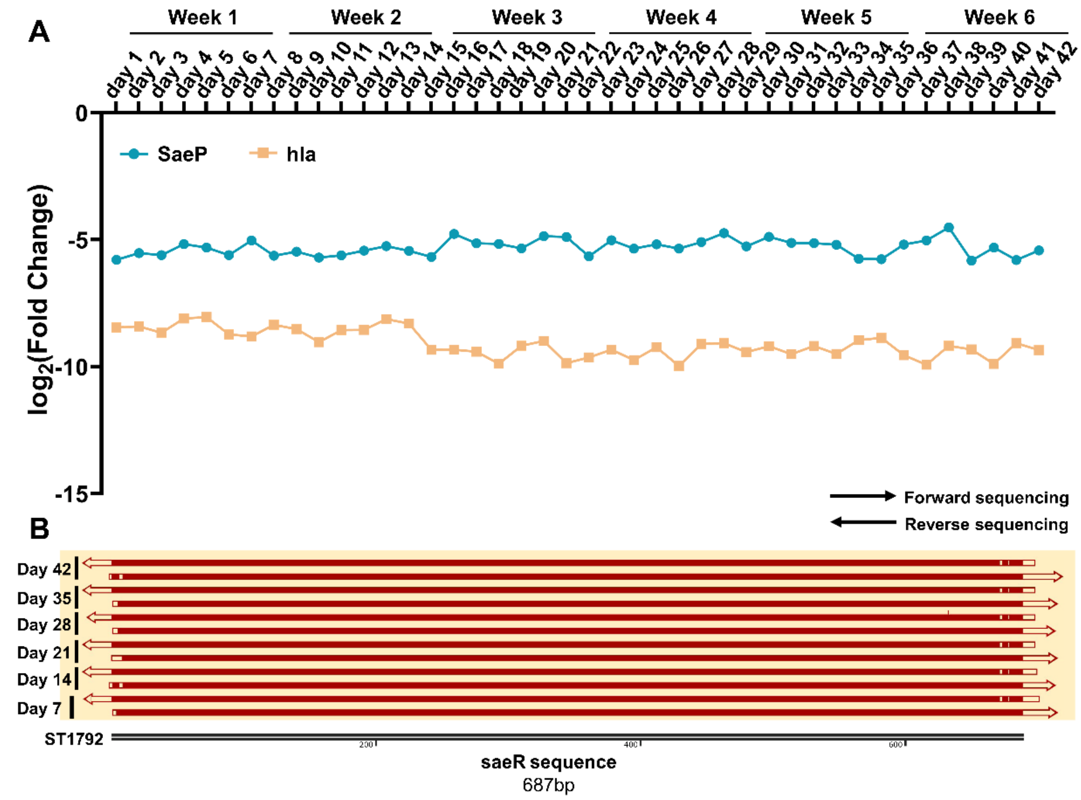

Supplementary Figure 19. *S. aureus* does not develop drug resistance to fenoprofen. (**A**) Fold change was the daily ratio of *saeP* and *hla* gene expression between fenoprofen-treated and control groups. Fenoprofen-treated group: *S. aureus* ST1792 was treated with fenoprofen (100 μM). Control group: *S. aureus* ST1792 was treated with DMSO at the same concentration (0.1%). Values are presented as mean of three independent experiments. (**B**) DNA was extracted from the bacteria on day 7, day 14, day 21, day 28, day 35, and day 42 respectively, and was performed PCR and sequenced of saeR. No spontaneous mutation of the saeR gene was observed.

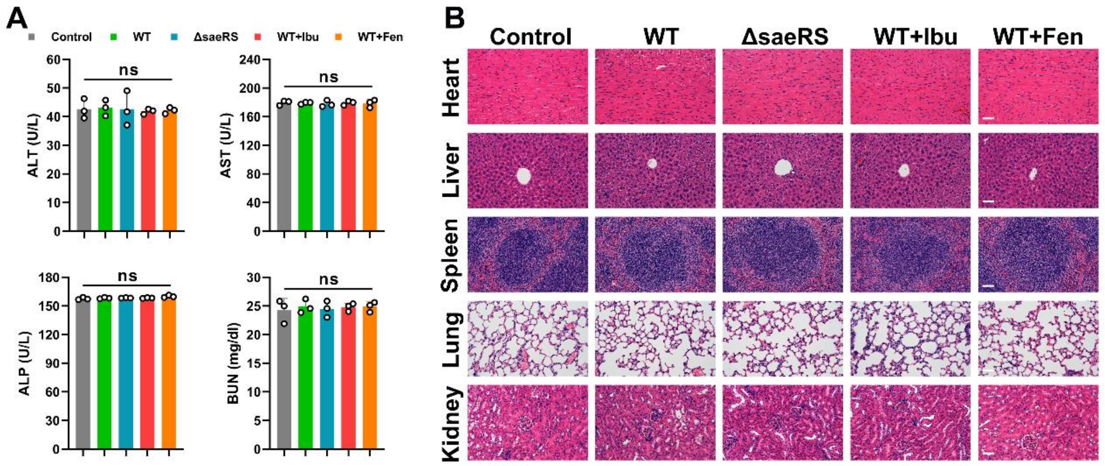

Supplementary Figure 20. The anti-infective dose of fenoprofen does not cause toxicity to mice and has excellent biocompatibility in vivo. (**A**) Analysis of renal and liver function (ALT, AST, ALP, BUN) in implant-associated infection mice treated with fenoprofen at day 7. The control group indicated healthy mice. (n = 3; data are presented as individual points). Data were analyzed by one-way ANOVA. (**B**) HE staining analysis of visceral organs of implant-associated infection mice treated with fenoprofen at day 7. Scale bar = 100 μm.

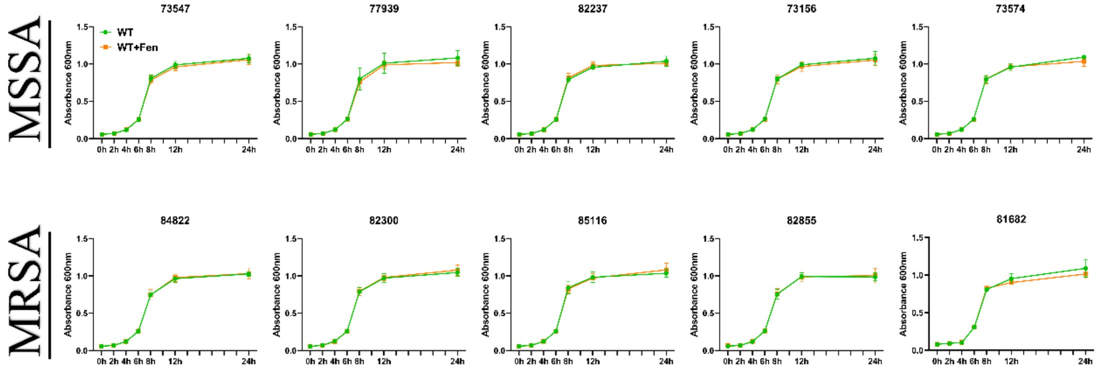

Supplementary Figure 21. Effects of fenoprofen on the growth ability of clinical strains. The 600nm absorbance of clinical strains (MSSA and MRSA) treated or untreated with fenoprofen were measured 0h, 2h, 4h, 6h, 8h, 12h and 24h. Each time point was repeated three times.

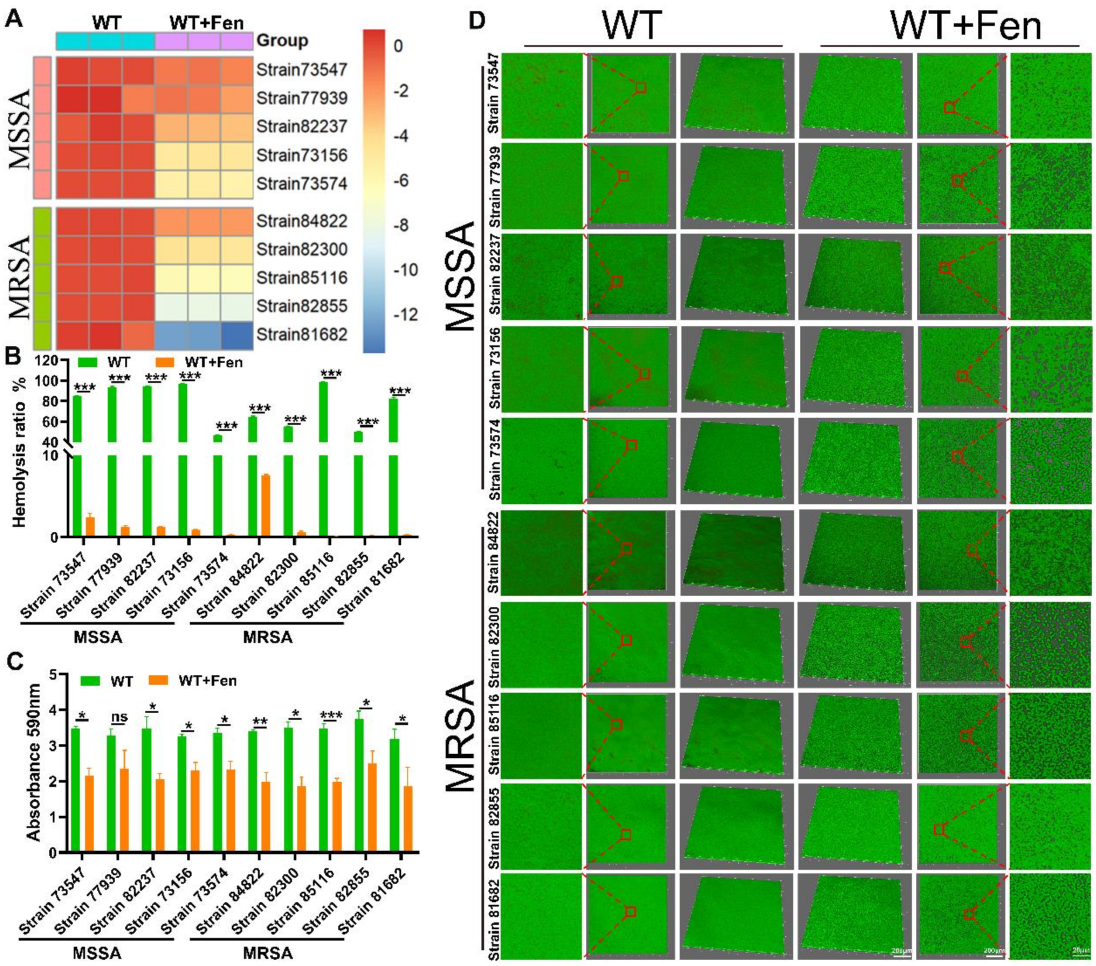

Supplementary Figure 22. Fenoprofen has excellent efficacy against clinical strains of *S. aureus*. (**A**) *Hla* expression levels of fenoprofen-treated and untreated *S. aureus* clinical strains. (**B**) Hemolysis test results of fenoprofen against *S. aureus* clinical strains (n=3). (**C**) Crystal violet staining data of biofilms in 96-well plates (n=3). (**D**) 3D reconstruction of biofilms formed by fenoprofen-treated and untreated *S. aureus* clinical strains. Scale bars=200 μm and 20 μm. All results are presented as the means ± SDs. *P<0.05, **P<0.01, ***P<0.001 and data were analyzed by two-tailed unpaired t tests (B and C).

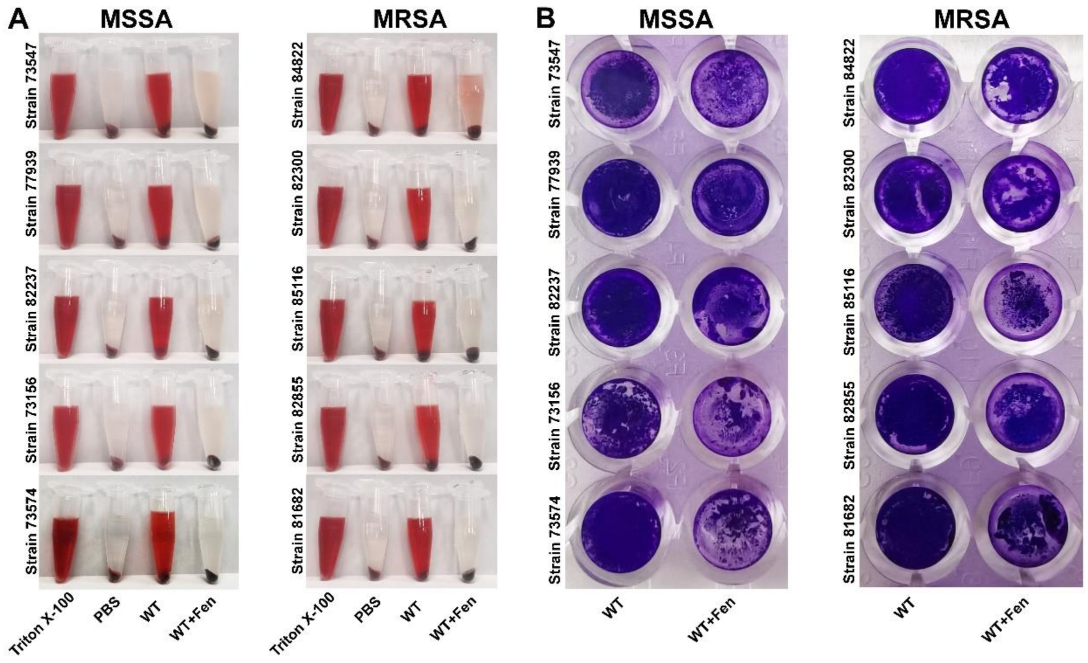

Supplementary Figure 23. Fenoprofen attenuated the hemolysis and biofilm formation abilities of *S. aureus* clinical strains. (**A**) Images of hemolysis test. *S. aureus* was treated with fenoprofen for 24h and then blood was added and cultured for 4 hours, 10000 RPM centrifuge for 10 min. (**B**) Images of crystal violet staining of biofilm in 96 well plate. Biofilms were grown in TSBg for 24h.

Supplementary Tables

Supplementary Table 1. Plasmids and strains used in this study.

Strains or plasmids	Relevant genotype or characteristic	Source
Plasmids		
pRN12	*E. coli-S. aureus* shuttle vector, Amp^r, Cm^r	[6]
pRN12-P1-GFP	*E. coli-S. aureus* shuttle cloning vector, carrying Sae P1 promoter and GFP segment, Amp^r, Cm^r	This study
pRN12-Phla-GFP	*E. coli-S. aureus* shuttle cloning vector, carrying *hla* promoter and GFP segment, Amp^r, Cm^r	This study
pRN12-sarA P1-GFP	*E. coli-S. aureus* shuttle cloning vector, carrying sarA P1 promoter and GFP segment, Amp^r, Cm^r	This study
pRN12-sarA P1-mcherry	E. coli-*S. aureus* shuttle cloning vector, carrying sarA P1 promoter and mcherry segment, Amp^r, Cm^r	This study
pET28a	Kmr, protein expression vector	Novagen
pET28a::saeR	pET28a derivative carrying saeR gene of *S. aureus*	This study

Staphylococcus aureus		
ST1792	MSSA, which was isolated from the prosthesis of a periprosthetic joint infection (PJI) patient	[7]
ST1792:: pRN12-P1-GFP	Wild-type *S. aureus* ST1792 carrying integration vector pRN12-P1-GFP	This study
ST1792:: pRN12-Phla-GFP	Wild-type *S. aureus* ST1792 carrying integration vector pRN12-Phla-GFP	This study
ST1792:: pRN12-sarA P1-GFP	Wild-type *S. aureus* ST1792 carrying integration vector pRN12-sarA P1-GFP	This study
ST1792:: pRN12-sarA P1-mcherry	Wild-type *S. aureus* ST1792 carrying integration vector pRN12-sarA P1-mcherry	This study
ST1792-lux	Lux fragment was inserted into *S. aureus* ST1792 genomic DNA	[7]
ST1792-Δ*saeRS*	*SaeRS* gene was knocked out from *S. aureus* ST1792	This study
ST1792-Δ*saeRS*:: pRN12-sarA P1-GFP	*SaeRS* mutant strain of *S. aureus* ST1792 carrying integration vector pRN12-sarA P1-GFP	This study
ST1792-Δ*saeRS*:: pRN12-sarA P1-mcherry	*SaeRS* mutant strain of *S. aureus* ST1792 carrying integration vector pRN12-sarA P1- mcherry	This study
USA300	A representative CA-MRSA isolate, which was first isolated in Los Angeles, Calif.	[8]
USA300:: pRN12-P1-GFP	Wild-type *S. aureus* USA300 carrying integration vector pRN12-P1-GFP	This study
USA300:: pRN12-Phla-GFP	Wild-type *S. aureus* USA300 carrying integration vector pRN12-Phla-GFP	This study
RN4220	Derivative of 8325-4 that accepts plasmids	Laboratory stock
ATCC 43300	MRSA, one of the laboratory standard strains of MRSA.	Laboratory stock
Strain 73547	MSSA, *S. aureus* clinical strain, which was isolated from the orthopedic infection patient	Laboratory stock
Strain 77939	MSSA, *S. aureus* clinical strain, which was isolated from the orthopedic infection patient	Laboratory stock
Strain 82237	MSSA, *S. aureus* clinical strain, which was isolated from the orthopedic infection patient	Laboratory stock

Strain 73156	MSSA, *S. aureus* clinical strain, which was isolated from the orthopedic infection patient	Laboratory stock
Strain 73574	MSSA, *S. aureus* clinical strain, which was isolated from the orthopedic infection patient	Laboratory stock
Strain 84822	MRSA, *S. aureus* clinical strain, which was isolated from the orthopedic infection patient	Laboratory stock
Strain 82300	MRSA, *S. aureus* clinical strain, which was isolated from the orthopedic infection patient	Laboratory stock
Strain 85116	MRSA, *S. aureus* clinical strain, which was isolated from the orthopedic infection patient	Laboratory stock
Strain 82855	MRSA, *S. aureus* clinical strain, which was isolated from the orthopedic infection patient	Laboratory stock
Strain 81682	MRSA, *S. aureus* clinical strain, which was isolated from the orthopedic infection patient	Laboratory stock
E. coli		
BL21(DE3)	F^- ompT hsdSB (rB^- mB^-) gal dcmmet (DE3)	Shengong, shanghai
DH5α	F-φ80 lac ZΔM15Δ (lacZYA-arg F) U169 endA1 recA1 hsdR17(rk^-,mk^+) supE44 λ-thi-1 gyrA96 relA1 phoA	Shengong, shanghai

Cm^r, chloroamphenicol resistance; Amp^r, ampicillin resistance.

Supplementary Table 2. Primers used in this study.

Gene	Primer direction	Primer sequence (5'→3')	reference
gyrB	Forward	GGTGGCGACTTTGATCTAGC	[9]
gyrB	Reverse	TTATACAACGGTGGCTGTGC	[9]
SaeR	Forward	CGTCCTCGTCACTTTGTTGA	This work
SaeR	Reverse	ATCGTGGATGATGAACA	This work
saeS	Forward	TGTTGCGCGAGTTCATTAGC	This work
saeS	Reverse	ACGGGTGGTTCTGGATTAGG	This work
saeP	Forward	TAATTTAGCGCCGCCGAAGA	This work
saeP	Reverse	TCGCAATGGTTGACTACGAT	This work
saeQ	Forward	TTATCATTCGTGTGGGTTCAGGTA	This work
saeQ	Reverse	TCCGCCCGTTAATTTTTCG	This work
WalR	Forward	AGTATGTCGTGAAGTGCGCA	This work

WalR	Reverse	AAGTTCGCTTTCACACGTGC	This work
WalK	Forward	AGCGTCGTGAATTTGTTGCC	This work
WalK	Reverse	TGGCGCAAGTTCCTCATCTT	This work
lytR	Forward	CTGCACATGACCAATACGCAG	This work
lytR	Reverse	TCGCCGACATATCATTCGCA	This work
lytS	Forward	TCCGAATTGCCCGCTTAGAG	This work
lytS	Reverse	ACTTTGCGTTTCGGCTTCAC	This work
GraR	Forward	TGGAACTTGGCGCAGATGAT	This work
GraR	Reverse	TCAACGACAGCATCTTGCCA	This work
GraS	Forward	CGAGGATTTACGTCAACGGC	This work
GraS	Reverse	TCACTTCCGACATGCGTTCA	This work
ArlR	Forward	TTCTGCGCCATTTACCGTCA	This work
ArlR	Reverse	TTGCTGGGCTTGATTACGGT	This work
ArlS	Forward	AATCACTCCAAATGCCAGCG	This work
ArlS	Reverse	CACCGTTATTTGACCGCGT	This work
srrA	Forward	AGTTGCCACCTGGATACCATC	This work
srrA	Reverse	CCATGAAGCAAGTAATGGCCA	This work
srrB	Forward	ACGCTGCAATAGGCTGAACT	This work
srrB	Reverse	TCGCTTGCCATTGTCCTTGA	This work
phoP	Forward	ATAGCCTAAGCCACGCACAG	This work
phoP	Reverse	GCAGAGTCATTACGAGAGAGCA	This work
phoR	Forward	TTGAACGTTGAGCCTTTGCC	This work
phoR	Reverse	TGGTGGGACAGGTCTTGGAT	This work
VraS	Forward	ACAGCAAAGCACGCATTTCT	This work
VraS	Reverse	TTTGCGGCAAGTATGATGCT	This work
VraR	Forward	ACGAACTGCATCGGCGATAT	This work
VraR	Reverse	TGGATGGTGTAGAAGCGACG	This work
AgrA	Forward	TCTCACAGACTCATTGCCCA	This work
AgrA	Reverse	TCACCGATGCATAGCAGTGT	This work
AgrC	Forward	ACCCTATCATTCGCGTTGCA	This work
AgrC	Reverse	ACCTAAACCACGACCTTCACC	This work
KdpE	Forward	ATGTTGATGAGCTTCGGGCA	This work
KdpE	Reverse	TGGAGCCAAAGTCGATGCTT	This work
KdpD	Forward	ACCAATTGGCACATCTCCGA	This work
KdpD	Reverse	ACGTCACATTTTCAACGGCG	This work
HssR	Forward	GCGTGTCGATATTGCAGTGG	This work
HssR	Reverse	AAACGCACGCTCTTTGTCAC	This work
HssS	Forward	TGGTGGTGCCATTGACATCG	This work
HssS	Reverse	AAACGTGCTTCAGCTTGTGG	This work
NreC	Forward	TTGGTAAGCTTCGACGCCAT	This work
NreC	Reverse	ATGATCACGCTGTTGTCCGT	This work
NreB	Forward	TGACGCTACCGCTGACTAAC	This work
NreB	Reverse	GCGCCATTTGGTAGCAGAAG	This work

hla	Forward	GGTATATGGCAATCAAC	This work
hla	Reverse	CTCGTTCGTATATTACATCT	This work
coa	Forward	CACAACCAGTTGCACAACCA	This work
coa	Reverse	GGCCGCTTTGTTCCATTGTT	This work
nuc	Forward	TGGTCCTGAAGCAAGTGCAT	This work
nuc	Reverse	AGCCAAGCCTTGACGAACTA	This work
efb	Forward	AGATGCGAGCGAAGGATACG	This work
efb	Reverse	TGGACGTGCACCATATTCGA	This work
splA	Forward	TCACTTTTGCTCCGTCTGCA	This work
splA	Reverse	CCGGAAAAGAAGACCTTGCG	This work
lukE	Forward	TTGCTGAACCTGTTGGACCA	This work
lukE	Reverse	TGGGGTGTTAAAGCAAACGA	This work
map	Forward	CCCATGTGCCACCTCTTCAT	This work
map	Reverse	AGCTGCAACCAAACCAGGTA	This work
hlgC	Forward	TCCAATCAGCCCCATCACTC	This work
hlgC	Reverse	ATTCGCTTTGACGCCCCATA	This work
fnbpB	Forward	GGTTGCTCAGTTGATGTCGC	This work
fnbpB	Reverse	GCTGCAGCATCGGAACAAAA	This work
fnbpA	Forward	ACCGCTATTTTGGCCACCTT	This work
fnbpA	Reverse	AAGCACAAGGACCAGTCGAG	This work
tst	Forward	ACAGCGTAGTTATGAGGGGT	This work
tst	Reverse	GGTGCACTGACAACTCCAGA	This work
aur	Forward	TTTAGAGCGCCTGACTGGTC	This work
aur	Reverse	TGGTGATGGTCGCACATTCA	This work
atlA	Forward	GGTGCAGTCGGTAACCCTAGAT	This work
atlA	Reverse	TGAACGTGCAAATGAAGCATAGT	This work
icaA	Forward	AGGTAAAGCCAACGCACTCA	This work
icaA	Reverse	AGCAAGTGTCTGACTTCGCT	This work
pRN12	Forward	GCTAGCGGATCCTCTAGAGTCG	This work
pRN12	Reverse	GGATCCCCGGGTACCTTAGGAGG	This work
saeP1	Forward	CGACTCTAGAGGATCCGCTAGCTTGGTACTTGTATTTAATCG	This work
saeP1	Reverse	CCTCCTAAGGTACCCGGGGATCCGTTGTGATAACAGCACCAGC	This work
Phla	Forward	CGACTCTAGAGGATCCGCTAGCAATTTTCTTATAATGCCTC	This work
Phla	Reverse	CCTAAGGTACCCGGGGATCCGATTTGAGGAAACAATAATCAATATGTC	This work
Phla (EMSA)	Forward	TATTAGATATTTCTATGTAATGGC	This work
Phla (EMSA)	Reverse	GCATTTCAATTTCGAGGGTTAGTC	This work

REFERENCES

1. Lipinski, C. A.; Lombardo, F.; Dominy, B. W.; Feeney, P. J., Experimental and computational approaches to estimate solubility and permeability in drug discovery and development settings. *Advanced drug delivery reviews* **2001,** *46* (1-3), 3-26.

2. Baell, J. B.; Holloway, G. A., New substructure filters for removal of pan assay interference compounds (PAINS) from screening libraries and for their exclusion in bioassays. *Journal of medicinal chemistry* **2010,** *53* (7), 2719-40.

3. Shelley, J. C.; Cholleti, A.; Frye, L. L.; Greenwood, J. R.; Timlin, M. R.; Uchimaya, M., Epik: a software program for pK(a) prediction and protonation state generation for drug-like molecules. *Journal of computer-aided molecular design* **2007,** *21* (12), 681-91.

4. Fan, X.; Zhang, X.; Zhu, Y.; Niu, L.; Teng, M.; Sun, B.; Li, X., Structure of the DNA-binding domain of the response regulator SaeR from Staphylococcus aureus. *Acta crystallographica. Section D, Biological crystallography* **2015,** *71* (Pt 8), 1768-76.

5. Friesner, R. A.; Banks, J. L.; Murphy, R. B.; Halgren, T. A.; Klicic, J. J.; Mainz, D. T.; Repasky, M. P.; Knoll, E. H.; Shelley, M.; Perry, J. K.; Shaw, D. E.; Francis, P.; Shenkin, P. S., Glide: a new approach for rapid, accurate docking and scoring. 1. Method and assessment of docking accuracy. *Journal of medicinal chemistry* **2004,** *47* (7), 1739-49.

6. de Jong, N. W.; van der Horst, T.; van Strijp, J. A.; Nijland, R., Fluorescent reporters for markerless genomic integration in Staphylococcus aureus. *Scientific reports* **2017,** *7*, 43889.

7. Guo, G.; Zhou, H.; Wang, Q.; Wang, J.; Tan, J.; Li, J.; Jin, P.; Shen, H., Nano-layered magnesium fluoride reservoirs on biomaterial surfaces strengthen polymorphonuclear leukocyte resistance to bacterial pathogens. *Nanoscale* **2017,** *9* (2), 875-892.

8. Diep, B. A.; Gill, S. R.; Chang, R. F.; Phan, T. H.; Chen, J. H.; Davidson, M. G.; Lin, F.; Lin, J.; Carleton, H. A.; Mongodin, E. F.; Sensabaugh, G. F.; Perdreau-Remington, F., Complete genome sequence of USA300, an epidemic clone of community-acquired meticillin-resistant Staphylococcus aureus. *Lancet (London, England)* **2006,** *367* (9512), 731-9.

9. Labandeira-Rey, M.; Couzon, F.; Boisset, S.; Brown, E. L.; Bes, M.; Benito, Y.; Barbu, E. M.; Vazquez, V.; Höök, M.; Etienne, J.; Vandenesch, F.; Bowden, M. G., Staphylococcus aureus Panton-Valentine leukocidin causes necrotizing pneumonia. *Science (New York, N.Y.)* **2007,** *315* (5815), 1130-3.
